# Private and External Costs and Benefits of Replacing High-Emitting Peaker Plants with Batteries

**DOI:** 10.1021/acs.est.2c09319

**Published:** 2023-03-14

**Authors:** Jason Porzio, Derek Wolfson, Maximilian Auffhammer, Corinne D. Scown

**Affiliations:** † Energy Analysis and Environmental Impacts Division, Lawrence Berkeley National Laboratory, Berkeley, California 94720, United States; ‡ Energy and Biosciences Institute, University of California, Berkeley, Berkeley, California 94720, United States; § Civil and Environmental Engineering Department, University of California, Berkeley, Berkeley, California 94720, United States; ∥ Department of Agricultural and Resource Economics, University of California, Berkeley, Berkeley, California 94720, United States; ⊥ National Bureau of Economic Research, Cambridge, Massachusetts 02138, United States; # Life Cycle, Economics and Agronomy Division, Joint BioEnergy Institute, Emeryville, California 94608, United States; ∇ Biological Systems and Engineering Division, Lawrence Berkeley National Laboratory, Berkeley, California 94720 United States

**Keywords:** Li-ion batteries, human health, air pollution, life-cycle assessment, electricity grid

## Abstract

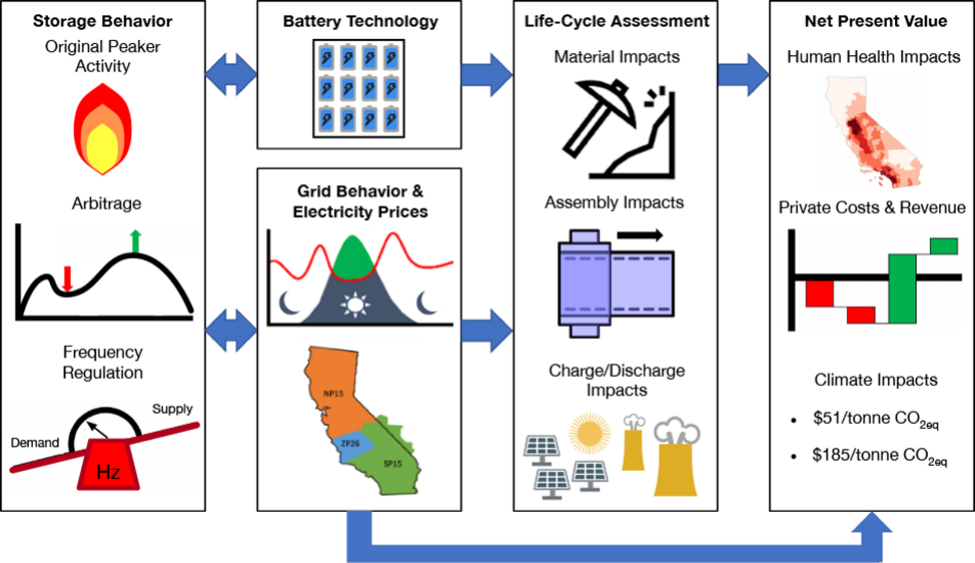

Falling costs of lithium-ion (Li-ion) batteries have made them attractive for grid-scale energy storage applications. Energy storage will become increasingly important as intermittent renewable generation and more frequent extreme weather events put stress on the electricity grid. Environmental groups across the United States are advocating for the replacement of the highest-emitting power plants, which run only at times of peak demand, with Li-ion battery systems. We analyze the life-cycle cost, climate, and human health impacts of replacing the 19 highest-emitting peaker plants in California with Li-ion battery energy storage systems (BESS). Our results show that designing Li-ion BESS to replace peaker plants puts them at an economic disadvantage, even if facilities are only sized to meet 95% of the original plants’ load events and are free to engage in arbitrage. However, five of 19 potential replacements do achieve a positive net present value after including monetized climate and human health impacts. These BESS cycle far less than typical front-of-the-meter batteries and rely on the frequency regulation market for most of their revenue. All projects offer net air pollution benefits but increase net greenhouse gas emissions due to electricity demand during charging and upstream emissions from battery manufacturing.

## Abstract 2

Incorporating air pollution-related health and climate impacts can alter the cost−benefit analysis for clean energy projects. This study explores the tradeoffs of replacing high-emitting peaker power plants with lithium-ion batteries.

## Introduction

The cost of lithium-ion (Li-ion) batteries has dropped dramatically in the last three decades, making them a competitive option for deployment in electric vehicles, household power management, and grid-scale energy storage.^1−5^ These battery energy storage systems (BESS) can help address the intermittency of renewable generation and the need for frequency regulation on the grid.^6−8^ Because Li-ion batteries offer fast ramping, they are well suited to mitigate the grid impacts of the “duck curve” in typical summer-peaking regions where renewable energy is plentiful during midday but less available during some of the highest-demand times (e.g., evening and early morning, although this timing may change with the emergence of new technologies like heat pumps).^9−12^ Properly operating Li-ion batteries do not emit local or global pollutants at the point of installation, which makes them an attractive replacement for high-emitting “peaker plants,” which are often located in disadvantaged communities and operate on hot days when ambient ozone concentrations are high.^13^ The practice of decommissioning peaker plants and installing BESS in their place has been hypothesized to generate significant benefits by reducing onsite air pollutant emissions and providing other revenue-generating grid services (e.g., grid stabilization).^14−17^

In California and New York, there are active requests for proposals to replace peaker plants with Li-ion BESS, and the first battery storage installations have already come online (Table S4).^18−22^ These facilities aim to earn revenue while avoiding peaker plant generation and its associated emissions. What remains unanswered is how total social costs (private and external) compare to total social benefits for these peaker replacement projects. In other words, if the goal is to reduce greenhouse gas (GHG) emissions and decrease the burden on human health, can these peaker plant replacement projects deliver on their promise? If so, what conditions are required to make the BESS installations economically attractive for profit-maximizing firms and society as a whole? To answer these questions, we evaluate the full life-cycle costs and air quality impacts of replacing California’s highest-emitting natural gas peaker plants with BESS. We explore how the net present value (NPV) is impacted by incorporating monetized human health benefits from avoided air emissions as well as revenue from arbitrage and grid services that BESS can provide.

## Materials and Methods

### Selection of Natural Gas Peaker Plants

**Figure 1:**
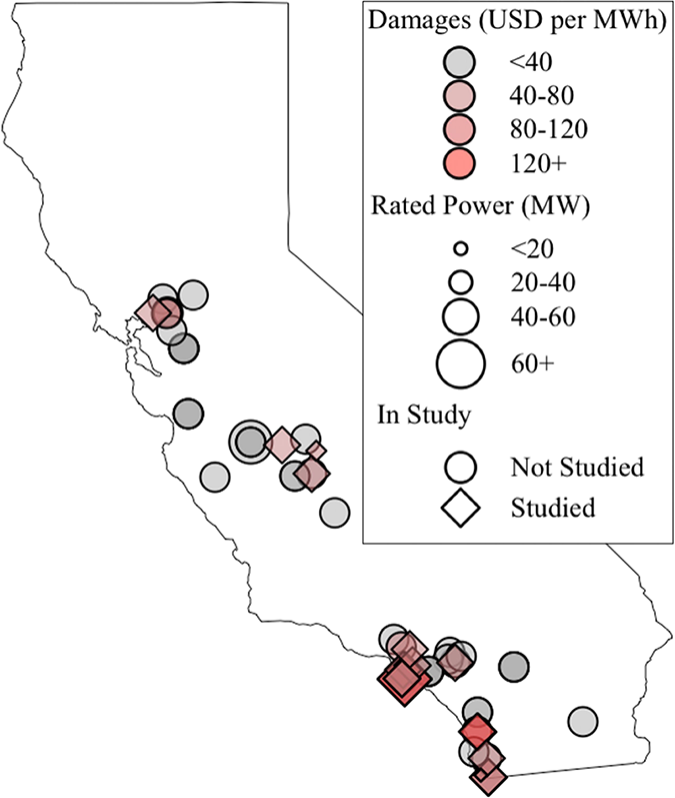
Map of natural gas peaker power plants in California. Each natural gas peaker plant is represented by one icon on the map. Color represents the monetized damages per MWh in USD caused by emissions from that plant between 2018 and 2020. Size represents the rated power of a power plant in MW. A circle indicates that replacement by BESS may be feasible but is not studied in the paper. An oblique square indicates that the impacts of replacement by BESS are studied for the natural gas peaker plant.

We focused our analysis on California because the state is home to the only completed peaker plant replacement project to-date, in addition to several BESS installations designed to reduce (but not eliminate) peaker activity, with large amounts of energy storage projects that are planned.^19−21^ Additionally, due to the high penetration of solar photovoltaics (PV) in California, the state is facing near-term grid impacts associated with the “duck curve” that must be mitigated through energy storage investments and/or fast-ramping power plants.^10,11^ To understand the economic attractiveness of BESS replacements for peakers, we selected a set of peaker plants currently operating across California and then modeled their hypothetical replacement. We began the process of selecting peaker plants by considering California’s 228 natural gas-fired power plants included in the EPA’s Continuous Emissions Monitoring Systems (CEMS); although California does have oil and diesel-fired generators, these plants are not large enough to be included in CEMS.^23^ Peakers were chosen for further analysis if they are in the top quintile of total air emission-related damages (monetized, including climate change and human health) per unit of energy output, have a maximum continuous output (a single generation event) under 1200 MWh, and are not a cogeneration facility. Climate damages were estimated based on the social cost of carbon, and human health damages were modeled using the Estimating Air pollution Social Impact Using Regression (EASIUR) model, as described in Procedure S7. The selection criteria yielded 19 generation facilities for hypothetical replacement. Figure 1 displays the location of all plants (selected and not selected) with maximum continuous output under 1200 MWh, their normalized climate and human health damages from stack emissions (CO_2_, NO_*X*_, SO_*X*_, and PM_2.5_), and rated power in MW. Upstream/life-cycle emissions were not included in the screening criteria used to select peaker plants for replacement. Operational data and stack emissions for natural gas combusting generators in California are from 2018 through 2020 and were obtained through CEMS.^23^ We assume that all modeled BESS are sited as close as possible to the corresponding offset peaker plants in order to reduce uncertainty with geographical market variance and infrastructure requirements. Additionally, each BESS is modeled independently, so the model does not consider any interactions that might occur if multiple peakers were simultaneously replaced with BESS.

### Battery Energy Storage System Sizing, Operation, and Upfront Costs

To understand the costs and net air pollutant emission impacts of installing BESS in place of peaker plants, we needed to identify locations, size each system appropriately based on the peaker it is replacing, and then simulate how the battery would be charged and discharged throughout each day. We assumed each new BESS will be located at the same site as the corresponding peaker plant it replaces and will not exceed the peaker’s maximum power output during charging or discharging. This avoids having to model additional potential costs associated with upgrading transmission and distribution infrastructure, which are outside the scope of this study. Additionally, we assumed that the BESS will have a four-hour discharge duration; while this represents the higher end of durations for front-of-the-meter BESS in the US,^24^ a four-hour duration is frequently used when studying peaker replacement capabilities and in rulemaking for California and New York.^25−27^ This assumption dictates that the power-to-energy ratios of all modeled BESS will be 0.25.

We used optimization to determine the minimum necessary rated energy storage capacity of the BESS based on how each peaker plant has historically operated. Unlike peaker plants, the BESS must be charged, and those charging decisions will impact the optimal sizing, facility economics, and emissions. The optimization program developed for this study considers the historical output of each natural gas peaker plant and local hourly electricity prices from 2018 through 2020, obtained through California Independent System Operator (CAISO) Open Access Same-time Information System (OASIS) and CEMS,^23,28^ to minimize the rated energy storage capacity of the BESS while simultaneously determining the charging decisions that minimize the cost of purchasing electricity. Several previously published studies used optimization to estimate profits earned during the operation of BESS;^29−32^ we used an approach most similar to the linear method outlined by Nguyen et al.,^33^ which reduces the computational requirements. The optimization model is described in greater detail in Procedure S1.

After determining the minimum necessary rated energy storage capacity, we determined the capacity fade (referred to here as degradation) that the Li-ion cells will experience during their operation. Battery systems for each BESS were then over-sized to ensure they could deliver a consistent level of service after compensating for this loss. Degradation is accounted for based on two separate mechanisms: degradation from cycling, and degradation from maintaining a state-of-charge over time (shelf-life degradation). Increasing the number of times the system is cycled and extending the length of time before the battery is replaced will both increase the required size of the battery system. We assume that all BESS will have a scheduled battery replacement midway through the facility’s lifespan. This assumption reflects expected market behavior, given the longer lifetimes of many system components relative to the Li-ion batteries themselves.^34−36^ The simulated charging and discharging behavior for peaker replacement and arbitrage behavior is used to determine the expected degradation. Further details of battery oversizing and degradation are presented in Procedure S8, S9, Tables S5, S6, and Figure S2.

**Figure 2:**
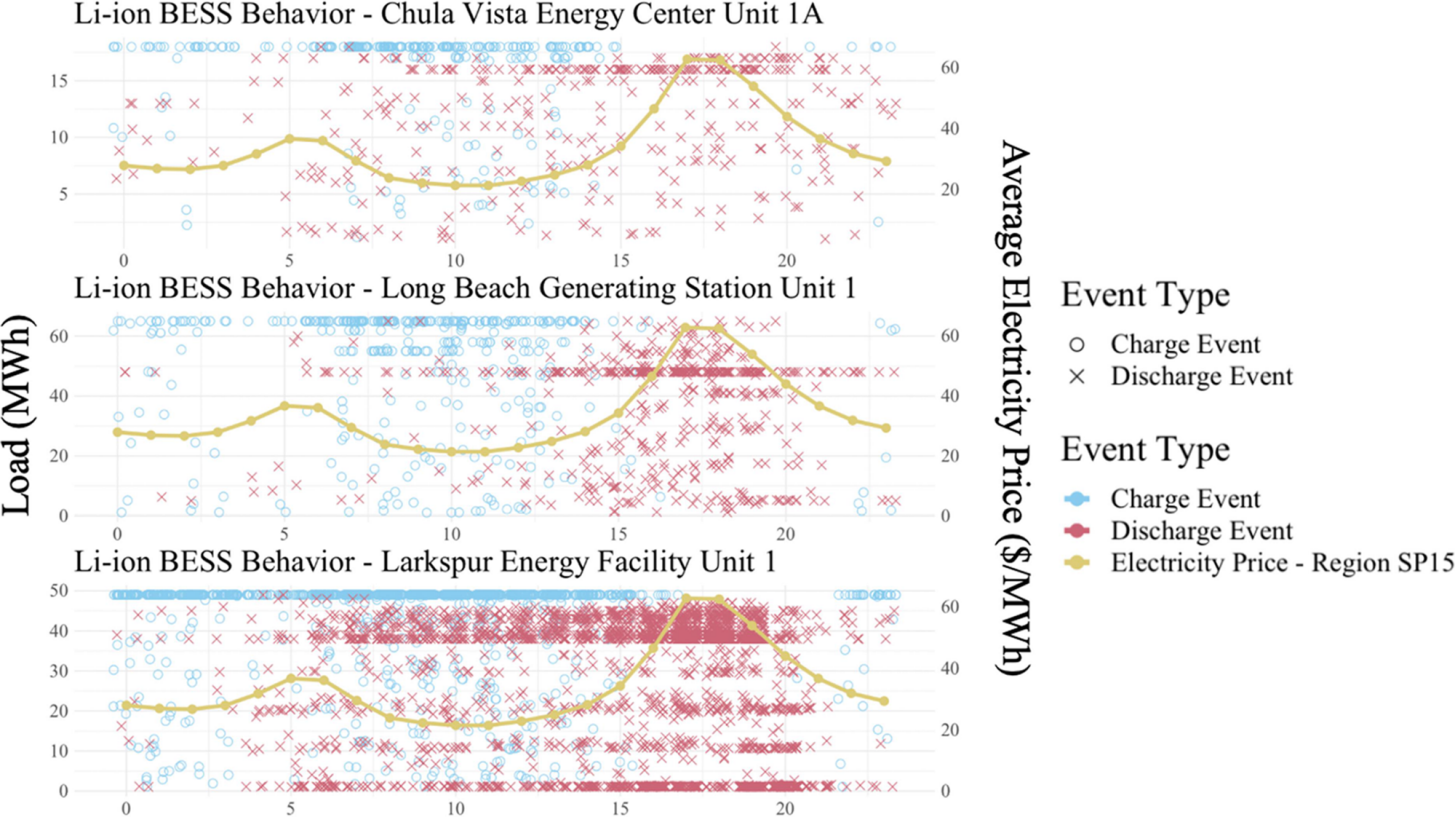
Charging behavior of selected BESS in 2018–2020 for peaker replacement considering electricity prices. The time of day and load (MWh) of each charge and discharge event from 2018 through 2020 is illustrated for three natural gas peaker plants. The optimized charging events are represented as blue circles. The fixed discharging events are represented as red x’s. Chula Vista Energy Center Unit 1A is the peaker plant with the least output of the studied peaker plants; Long Beach Generating Station Unit 1 has the median output; and Larkspur Energy Facility Unit 1 has the maximum output. The average electricity price at each time of day from 2018 to 2020 is plotted on the second axis to visualize the relationship between charging/discharging events and electricity price. Figure S3 visualizes the state of charge of the BESS offsetting Long Beach Generating Station 1 for two example weeks to further visualize behavior.

Figure 2 visualizes the optimized charging behavior of three example BESS for peaker replacement only, each replacing a different natural gas peaker plant representing the minimum (Chula Vista Energy Center Unit 1A), median (Long Beach Generating Station Unit 1), and maximum (Larkspur Energy Facility Unit 1) annual electrical generation of all peaker plants included in this study. The number of full charge–discharge cycle-equivalents required for peaker replacement varies widely by facility, with a high of approximately 62 cycles/year, a low of around 8, and an average across all facilities of 27 (Table S7). The charging times and loads determined by the optimization align with the expected behavior of an energy storage system, charging mostly during the day and early morning when electricity is cheapest. Exceptions to this expected behavior are due to daily variation in electricity price and peaker output. Large periods of continuous output may require charging at nonideal hours in order to store enough electricity to fully meet the required load. This is more common in plants with greater energy throughput, such as the Larkspur facility.

Using the optimal BESS sizing for each peaker replacement system as an input, we constructed a detailed technoeconomic model to quantify the private costs associated with the installation and operation of each BESS, using a bottom-up method similar to that of Feldman et al.,^37^ which is further documented in Procedure S4, Figure S4, Tables S8, S10, and S11. The initial cost results suggest that sizing BESS to fully replace natural gas peaker plants would require rated capacities well beyond what could be considered economically feasible. A first, albeit somewhat obvious, finding of this research is that building BESS to fully replace peaker plants will result in massive capital expenditures (CapEx) and insufficient revenue to compensate for those costs. However, if a BESS is instead sized to meet the 95th percentile load event for each peaker plant (by hours of continuous generation), the required rated capacity decreases by nearly 80% in some cases. Other strategies or infrastructure will be required to supply the energy otherwise provided during the largest fifth percentile of load events served by natural gas peaker plants (roughly 19% of the current peaker output on average), such as demand response measures.^38−40^ For example, a cell phone alert from the California Governor’s Office of Emergency Services sent during a recent heat wave prompted a 1.2 GW drop in demand in a span of just 5 min.^41^ The relationship between BESS sizing and the fraction of peaker plant activity avoided is further explored in Procedure S2 which illustrates the BESS size required to offset varying percentiles of natural gas peaker plant activity.

### Potential for Arbitrage and Grid Services

While the hypothetical BESS studied here are sized and operated based on the need for peaker replacement, operators would be free to take advantage of other revenue-generating activities through arbitrage and the provision of grid services. BESS can engage in a variety of revenue-generating activities, and based on available information on the size and value of these markets, we identified arbitrage and frequency regulation as the most attractive options in the near term.^6,42−45^ We determined the revenue and emission impacts associated with arbitrage using a similar optimization approach to that previously described for predicting charging and discharging behavior (described in Procedure S3). In addition to arbitrage, providing grid services can serve as a source of substantial revenue for BESS.

Within the grid services that BESS are well positioned to provide, participation in frequency regulation markets offers a particularly large potential source of revenue for BESS.^46,47^ We model the revenue from frequency regulation as three main components in accordance with Xu et al.:^48^ capacity revenue, mileage revenue, and fast regulation revenue. Each component is further broken into an individual component for upward and downward mileage. Capacity revenue is modeled as the BESS available capacity for frequency regulation multiplied by the hourly frequency regulation capacity clearing price. Mileage revenue is modeled as the BESS available capacity for frequency regulation, multiplied by the hourly percentage of that capacity that is called on by CAISO, the hourly accuracy score, and the hourly mileage clearing price. The hourly frequency regulation capacity clearing price, the hourly percentage of called capacity, the hourly accuracy score, and the hourly mileage clearing price are sourced from CAISO for the years 2018 through 2020 modeled in this study. Additionally, while some independent system operators have an additional market minute regulation activity (referred to as fast regulation in this study), CAISO does not have a market for this service, so this component is omitted from modeling. Furthermore, we assume that frequency regulation and mileage cannot occur during arbitrage or peaker replacement to avoid conflicts with available capacity. Additional modeling details are available in Procedure S4.

In many instances, profits from frequency regulation exceed the profits from arbitrage in the same period (Table S12), yet our analysis prioritizes arbitrage over frequency regulation. This choice is based on the small size of the frequency regulation market and high likelihood that arbitrage will be more common in the future as the frequency regulation market becomes saturated.^43,46,47,49^Table S13 illustrates this point by comparing total electricity charged and discharged by batteries in California with the total frequency regulation market sizes (up and down).

## Results and Discussion

### Net Present Value of Battery Energy Storage Systems

To understand whether replacing peaker plants in California with BESS is profitable, we explored a range of scenarios and calculated the NPV for each. To capture differences among Li-ion cathode materials, we explored three alternatives: LiFePO_4_ (LFP), LiNi_*x*_Co_*y*_Al_*z*_O_2_ (NCA), and LiNi_*x*_Mn_*y*_Co_*z*_O_2_ (NMC). We assigned a normalized price per kWh and set of degradation characteristics to each battery type, representing current prices and performance. Each battery is sized for a four-hour discharge duration. The system lifetime was varied between 15 and 20 years, with battery replacement occurring at 7.5 and 10 years, respectively (conservatively assuming battery prices remain constant). We performed upfront system sizing with respect to the battery replacement timeline through the methods outlined in Procedure S1 as well as the details outlined in Procedure S8, S9, Tables S5, S6, and Figure S2. We used the federally mandated social cost of carbon of 51 USD per metric ton of CO_2eq_ emitted in 2020^50^ as well as a higher social cost of carbon of 185 USD per metric ton of CO_2eq_ from Rennert et al.^51^ and included monetized human health damages from pollutants that form secondary fine particulate matter: primarily NO_*X*_. We explored three different discount rates: 3, 5, and 7% and applied these rates to both private costs/revenues and changes to monetized climate and human health damages. Additionally, the analysis includes operations and maintenance (O&M) costs, which entail replacement of heating, ventilation, and air conditioning (HVAC) equipment and other components with limited lifespans. Separate from the scenarios discussed here, we capture uncertainty in all other cost and design parameters using probability distributions (Table S8) and Monte Carlo simulations. The NPV of all BESS across all scenarios is presented in Figures S5–S10.

**Figure 3:**
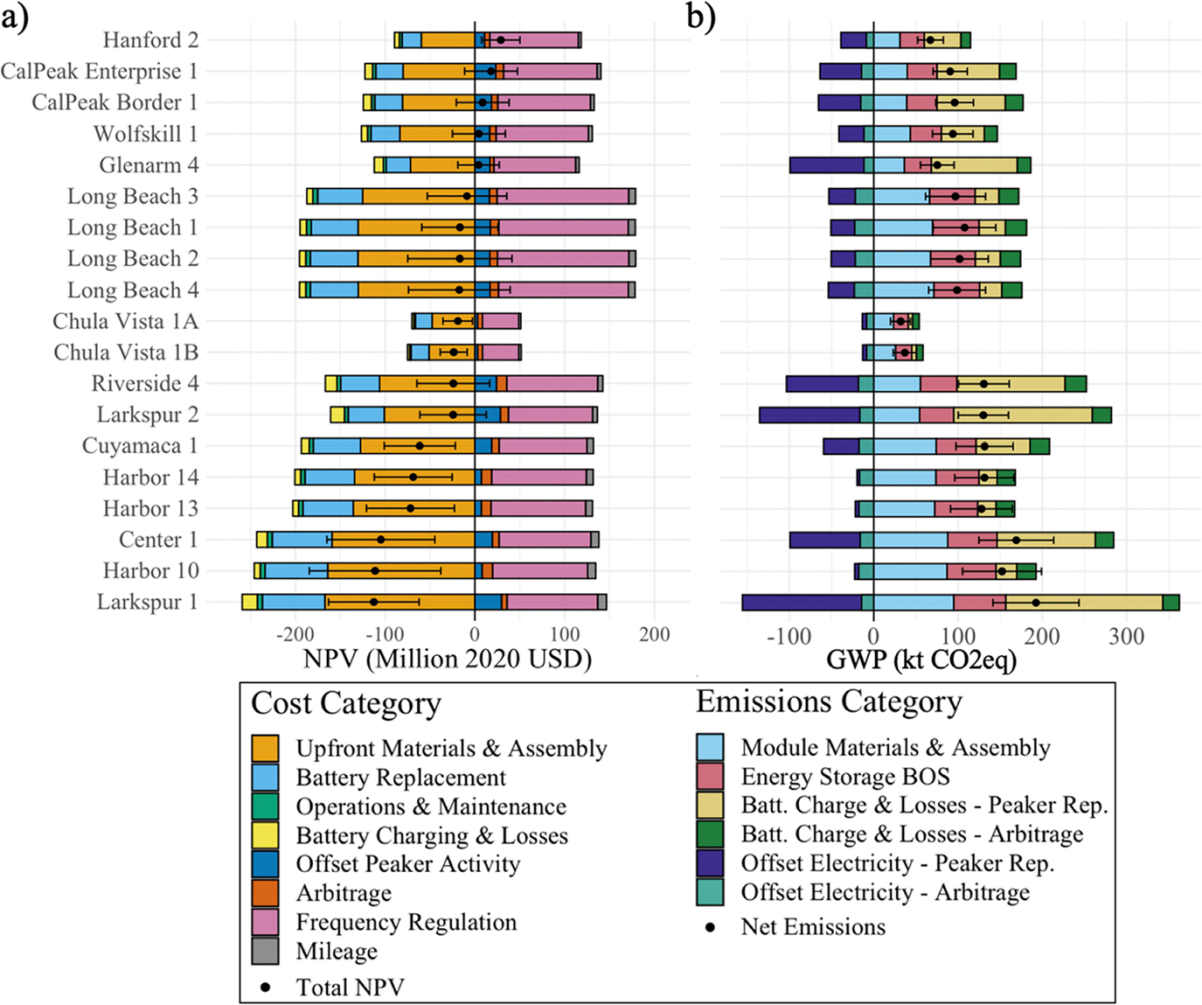
Net present value and global warming potential of BESS replacing natural gas peaker plants. Figure 3 illustrates the (a) NPV and (b) global warming potential of all the BESS explored for the scenario described, and breaks down the sources of costs and revenues by category. The NPVs and emissions are presented, as well as the uncertainty at two standard deviations, determined through Monte Carlo Simulation with 500 model runs. These results represent an LFP cathode with a battery replacement occurring after 10 years, a total facility lifetime of 20 years, and discount rate of 3%.

Figure 3 presents the NPV and net 100-year global warming potential (GWP) for each of the BESS replacing the 19 natural gas peaker plants considered in the study. These results include a LFP cathode with a replacement battery at 10 years, a total project lifespan of 20 years, and a discount rate of 3%. A more detailed breakdown of life-cycle GWP for the Long Beach 1 facility (a representative average case) is presented in Figures S11a and S11b, and the impact of changing design parameters and discount rates is discussed in the Sensitivity Analysis section. In 14 of the 19 hypothetical peaker replacements shown in Figure 3a, the expected total NPV falls below zero, while 5 have expected NPVs above zero. In 10 of the total projects presented, the uncertainty around the total NPV spans both negative and positive values, indicating that some of these BESS could be viable, particularly if Li-ion battery costs continue to fall. However, these results rely on current market values for frequency regulation, which may also fall as more BESS come online and saturate the market.

**Figure 4:**
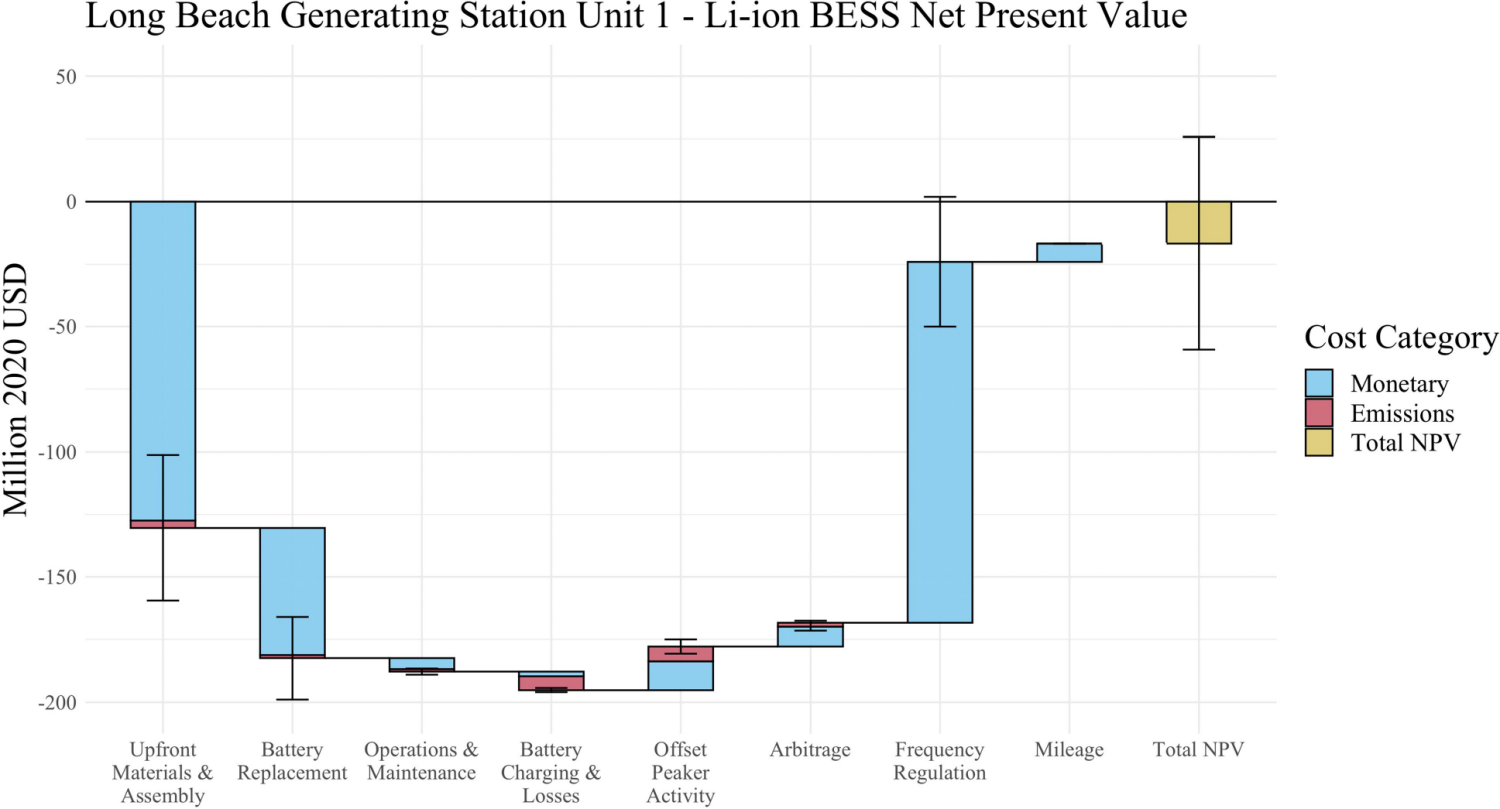
Net present value of BESS replacing Long Beach Generation Station Unit 1. Figure 4 illustrates the NPV of the BESS replacing Long Beach Generating Station Unit 1 and breaks down the sources of costs and revenues by category, additionally specifying the impact from monetary and environmental sources per category. Error bars represent two standard deviations.

Figure 4 provides a more detailed breakdown of the NPV for a single BESS, distinguishing between the private costs and revenue, as well as the monetized emissions impacts. The bars labeled “monetary” represent the private revenues and costs associated with building and operating the BESS. The emissions cost bars represent the monetized human health damages and climate damages resulting from the induced electricity generation due to battery charging. Emission offsets are modeled as the avoided damages to human health and the climate from electrical generation that the battery displaces when it is discharging. The remaining peaker plant activity that cannot be economically replaced with the BESS (any event with a greater energy demand than the 95th percentile peaker event) is not included as either a cost or benefit. Further details are provided in Procedure S7.

As shown in Figures 3a and 4, the monetary upfront and battery replacement costs represent the two largest costs across all BESS. The costs associated with both O&M and battery charging and losses are near negligible in comparison. Frequency regulation is the dominant source of revenue, despite the fact that we model the BESS to prioritize arbitrage whenever it is profitable. The other revenue-generating activities offsetting peaker activity, arbitrage, and mileage offer relatively small economic revenue streams compared to the total system cost. Prior studies have also emphasized the near-term profitability of ancillary service markets relative to arbitrage when choosing how to operate energy storage systems.^43,44,52^ The results in Figure 3a highlight that, while the key cost and revenue drivers remain consistent across all facility designs, the relative breakdown of costs and revenues for each BESS do vary. This variation suggests that some peaker plant replacement projects can be prioritized based on system characteristics that lead to more profitable BESS.

The two largest costs (upfront materials and assembly and battery replacement) are dictated by the BESS storage capacity required to meet the 95th percentile load event of the natural gas peaker plant being replaced (see Figure S1 and Table S9). Plants that historically have required frequent extended, continuous generation must be replaced with larger BESS, often with a rated power much greater than that of the peaker plant (Table S7), in part because of the degradation the batteries will experience over their lifetime. In contrast, the potential revenue from frequency regulation is dictated by the maximum power output of the BESS. In this study, the maximum power output for each BESS when it is operating is capped at the rated power of the replaced natural gas peaker plant. This prevents the model from inadvertently exceeding the capacity of the local grid infrastructure. However, the BESS can have a rated power greater than this if needed to ensure adequate storage capacity while maintaining a power-to-energy ratio of 0.25. Ultimately, a BESS will have a higher total NPV if the natural gas peaker plant being replaced has a relatively high rated power, yet is rarely called upon for extended, continuous generation.

While frequency regulation represents the greatest near-term source of revenue for all BESS, the future of this revenue stream is uncertain. Frequency regulation represents a small, fairly localized market.^43,46,47,49^ Given the forecasted growth of grid-connected energy storage in California,^19^ the value of frequency regulation will likely decrease over time. A key question is how this may be counterbalanced by anticipated reductions in battery costs.

In this study, the prices of replaced Li-ion cells are held constant at current market prices. However, many forecasts suggest that Li-ion cell prices will decrease,^1,53−55^ meaning the cost of battery replacements may be lower than what is modeled here. The degree of this potential price reduction is highly variable on how the Li-ion technology develops, especially since constant learning is not guaranteed.^56^ Technological learning for Li-ion batteries can drive prices lower, while material shortages and supply chain challenges for Li-ion cells may counterbalance some of these improvements.^57−59^ If the US Department of Energy’s $60/kWh target for Li-ion modules^60^ is reached in advance of when battery replacement occurs for the facilities in Figure 3, nine of the 19 BESS explored will have a positive NPV (as opposed to 5, based on current Li-ion battery prices).

### Global Warming Potential of Battery Energy Storage Systems

Figure 3b presents the life-cycle GWP of BESS in the previously described scenario. We conservatively assumed no recycling of Li-ion cells given the current challenges with Li-ion recycling supply chains.^61,62^ For perspective, a prior study estimated that recycling could save approximately one quarter of the Li-ion batteries’ GHG footprint, although results vary by the cathode material and recycling process.^63^ Future uncertainty in cell manufacturing and other energy storage components were captured in a Monte Carlo analysis. Probability distributions for input parameters are provided in Table S14. The life-cycle GWP for all plants across all scenarios is presented in Figures S12–S14.

The GWP for all BESS examined is net positive (based on the current grid mix), as illustrated in Figure 3b, meaning that system-wide life-cycle GHG emissions increase relative to the counterfactual case in which the peaker plant continues to operate and no BESS is installed. There are two reasons for this: first, the embedded emissions associated with the BESS and its eventual replacement are substantial and second, the replacement of peaker plant activity and engagement in arbitrage induce more GHG emissions at power plants elsewhere on the grid during BESS charging than what is saved during discharging. This result is not without precedent; Craig et al.^64^ found that grid-scale electricity storage would increase system-wide CO_2_ emissions for Electricity Reliability Council of Texas (ERCOT) in the very near-term, based on the outputs of their economic dispatch model. Our results for California echo this finding: with the current grid, charging can induce additional fossil-based generation, particularly when excess solar capacity is not available. Our modeling approach, described in Procedure S7, captures this behavior and estimates the impact on GWP from this induced thermal generation. Our modeling does not consider how the availability of storage may impact capacity expansion in the long run. As demonstrated by Bistline and Young,^65^ the availability of grid-scale battery systems can influence future investments in generating capacity and infrastructure, although the effects may increase or decrease emissions. Finally, energy losses attributable to the Li-ion cells and the balance-of-systems components such as HVAC translate to a round-trip efficiency ranging from 80 to 95%, meaning the battery consumes more electricity during charging than it supplies during discharging.

One may reasonably expect the impact of battery charging and discharging on GWP to be larger than what is shown in Figure 3b. When not replacing peaker plant activity, the optimization model allows each BESS to engage in arbitrage whenever it is profitable (accounting for electricity prices and battery degradation). However, as shown in Table S7, this occurs infrequently (an average of 8 cycles per year for LFP BESS). Replacing peaker plant activity requires more cycles (average of 27 across all BESS in this study). From our analysis, we determined that each BESS would likely spend the majority of the year participating in frequency regulation, which is the most profitable strategy but adds a negligible number of cycles and little to no emissions benefits. However, BESS installed for different use cases are reported to cycle more frequently. For example, a 2020 IHS report that sampled eight projects, with an average rated power of under 20 MW (considerably smaller than the BESS modeled here which have an average rated power of 97 MW) over a period of 1 to 5 years reported that the BESS cycled an equivalent of 251 times per year on average, with a minimum of around 75 and a maximum over 450.^66^

While it may be possible to achieve greater avoided emissions from offset electricity—and potentially a negative net GWP—through intentional system behavior and arbitrage,^34,67−69^ this behavior is not achievable in any profit-maximizing peaker replacement scenarios explored (in the context of the 2018–2020 grid) and may lead to significantly reduced revenues and increased costs associated with battery sizing due to higher degradation from cycling.

### Sensitivity Analysis

BESS design and input parameters for the cash flow analysis are likely to change as technology and market conditions evolve. The LFP cathode chemistry (shown in Figures 3 and 4) results in the most profitable BESS due to its reduced cell price, and it also results in lower life-cycle GWP because it avoids the need for cobalt, nickel, and manganese. Five of 19 BESS had a positive expected NPV when modeled with an LFP cathode, a 3% discount rate, 20-year lifespan, and social cost of carbon of 51 USD per metric ton of CO_2eq_. Similarly, five of the 19 BESS also had a positive expected NPV when modeled with an NCA cathode but had a lower average NPV across all 19 plants (−33 million 2020 USD versus −31 million 2020 USD for LFP cathodes). Only one BESS had a positive expected NPV with the NMC cathode. The impact of different cathode materials on NPV is provided in Figures S5–S10.

Altering the lifespan of the entire BESS facility can also substantially impact the NPV. Our modeling approach assumes a single battery replacement will occur midway through the lifespan of the BESS. The battery system is sized to deliver a consistent level of service, accounting for capacity fade from cycling and shelf-life degradation that will occur over half of the total BESS facility’s lifespan. Shortening the battery replacement time from 10 to 7.5 years (total BESS lifespan from 20 to 15 years) will require a smaller battery system to maintain a consistent level of service and, thus, CapEx decreases. However, decreasing the lifespan of the BESS also reduces the revenue earned while it is in service. In all scenarios explored, the revenue earned during a longer battery lifetime (replacement at 10 years, total BESS lifetime of 20 years) outweighed the increased CapEx. Specifically, at a 3% discount rate and a social cost of carbon of 51 USD per metric ton of CO_2eq_, going from a BESS lifespan of 15 to 20 years, the number of BESS with a positive NPV increased from 2 to 5 for the LFP and NCA cathode and 0 to 1 for the NMC cathode chemistry. The impact of different lifespans on NPV is demonstrated in Figures S5–S10. However, increasing lifespan is also associated with increasing life-cycle GWP, as more materials are required for the larger battery capacity, as shown in Figures S12–S14.

Varying discount rates also affects the NPV. While increasing the discount rate will lower the present value of the future battery replacement cost, it will also lower the value of future revenues. In the scenarios explored, increasing the discount rates slightly decreased the NPV of all BESS. For example, when modeled with an LFP cathode chemistry, a 20-year lifespan, and a social cost of carbon of 51 USD per metric ton of CO_2eq_, increasing the discount rate from 3 to 7% decreased the number of BESS with a positive NPV from 5 to 2. Figures S5–S10 visualize the impacts of changing discount rates on NPV.

To understand the impact of an elevated social cost of carbon, scenarios were performed with a cost of 185 USD per metric ton of CO_2eq_ emitted in 2020. This increased the upfront environmental costs associated with battery production as well as increasing the costs and benefits of battery operation. The cumulative impact is a net decrease in NPV across all scenarios because all BESS evaluated resulted in net positive life-cycle GWP. For example, when modeled with an LFP cathode chemistry, a 20-year lifespan, and a 3% discount rate, increasing the social cost of carbon from 51 to 185 USD per metric ton of CO_2eq_ caused the number of BESS with positive NPV to remain the same, but the average NPV decreased from −31 million 2020 USD to −36 million 2020 USD. Figures S5–S10 visualize the increasing the social cost of carbon on NPV.

## Discussion

Analyzing Li-ion BESS as replacements for natural gas peaker plants reveals several insights, some of which have implications for all front-of-the-meter battery storage. First, sizing BESS to fully replace the service provided by natural gas-fired peaker plants is unlikely to be economically viable. Instead, sizing each BESS to serve all but approximately the top fifth percentile of load events (appropriate threshold may vary by facility) dramatically reduces the required storage capacity and, thus, CapEx, while still meeting 81% of load on average (Table S9). This result highlights the continued need for demand-response^38−40^ and potentially mobile battery storage that can be called upon during extreme heat and other exceptional circumstances.^70^

Based on California’s current electricity market, BESS sized to meet the 95th percentile of loads served by natural gas peaker plants can achieve a positive NPV, but only if the value of frequency regulation does not decline. The BESS most likely to be profitable are those with LFP cathodes replacing large natural gas peaker plants that do not output large quantities of energy frequently and continuously, since most profits come from slack capacity sold in the frequency regulation market. Arbitrage, in contrast, is only a small contributor to total revenue. These findings are consistent with prior studies.^43,44,71^ However, given the limited size of the frequency regulation market and the forecasted growth of energy storage in California, the value of frequency regulation may decrease in the future.^43,46,47,49^ A remaining question is whether the social benefits of energy storage can compensate for the declining value of frequency regulation. Additionally, peaker plants can place a disproportionate environmental burden on historically marginalized groups.^72−74^ For example, the Hanford 2 peaker plant sits in a census tract where the PM_2.5_ concentrations are in the 99th percentile for the United States and nearly half of the population is Hispanic or Latino.^75^ Based on our analysis, this plant is potentially the most profitable target for replacement with a BESS. The community around the Wolfskill 1 facility averages PM_2.5_ concentrations in the top 95th percentile for the nation and is also approximately half Hispanic or Latino.^75^ Combining an understanding of the economics of replacement, alongside data on the distributional impacts of each plant’s emissions, can be a compelling strategy for replacing high-emitting plants.

The BESS scenarios evaluated in this study yielded small monetized climate and human health impacts relative to the private costs and benefits. While replacing peaker power plants does reduce air quality-related health damages in surrounding communities, the profit-maximizing behavior for the BESS we modeled also increased life-cycle GHG emissions once the embodied emissions in the BESS were accounted for. It may be possible to achieve a net zero or negative GWP through an intentional arbitrage strategy to reduce emissions^68,69^ and the installation of additional renewable resources on the grid can increase the likelihood that BESS will offer net environmental benefits.^34,67^ In the near-term, optimizing for emissions reductions would be less profitable due to increased cycling and reduced availability for frequency regulation.

Future prices of Li-ion cells and the evolution of electricity markets are critical to increasing the NPVs of BESS. If the value of frequency regulation does indeed decrease over time, battery costs must decrease and revenue from arbitrage must increase to maintain or increase NPVs. If the US Department of Energy target price for Li-ion modules, $60/kWh,^60^ is reached as battery replacement occurs for the scenario in Figure 3a, then nine of the 19 BESS explored will have a positive NPV, instead of 5. However, achieving this price reduction in 7.5 to 10 years will require learning rates much higher than the recent average learning rates for Li-ion cells.^1^ The rate at which Li-ion battery prices will decrease in the future is highly uncertain.^76^ Additionally, we modeled the future operation of BESS assuming electricity prices will remain at 2018 to 2020 prices over the next 15 to 20. This will almost certainly not be the case. In reality, transmission investments, new generation capacity, shifting demand, and changes in utility rate structures will influence the NPVs of BESS.

While we modeled realistic conditions for Li-ion energy storage aimed at replacing peaker plants in California, there may be a greater monetary value of storage technologies in other scenarios. In particular, some regions rely on coal combustion to meet peak demand, and combining BESS with renewable generation resources may further increase profitability while avoiding emissions associated with electricity generation.^34,67,77−79^ Other energy storage technologies like redox flow batteries or hydrogen storage may ultimately prove to be better suited for peaker replacement as they mature.^80−85^ Additionally, uncertainty in near-term energy supply may cause variation in market sizes and structure, altering future revenues.^86,87^ Finally, the modeled NPVs also do not capture the monetized human health impacts tied to rolling blackouts or prolonged outages,^88−90^ as well as the nonhealth community impacts associated with the removal of natural gas combustion peaker plants.^91^ Including these considerations may increase the value of BESS, especially since greater renewable integration and worsening effects from climate change increase the variability of electricity supply and demand.^92−96^

## Supporting Information Available

Additional modeling procedures, tables, and figures outlining specific methods and data used to evaluate technology costs and benefits (PDF)
