# Supplementary material for: Private and External Costs and Benefits of Replacing High-Emitting Peaker Plants with Batteries
Source: Environ Sci Technol. 2023 Mar 14;57(12):4992–5002. doi: 10.1021/acs.est.2c09319 (PMC10061926; doi:10.1021/acs.est.2c09319)
Supplement: Supplementary file 1 — es2c09319_si_001.pdf [file es2c09319_si_001.pdf]

Supporting Information

Private and External Costs and Benefits of Replacing High-Emitting Peaker Plants with Batteries

Jason Porzio[1,2,3], Derek Wolfson[4], Maximilian Auffhammer[1,4,5]*, Corinne D. Scown[1,2,6,7]*

[1] Energy Analysis & Environmental Impacts Division, Lawrence Berkeley, National Laboratory, Berkeley, CA 94720 United States
[2] Energy & Biosciences Institute, University of California, Berkeley, Berkeley, CA 94720 United States
[3] Civil & Environmental Engineering Department, University of California, Berkeley, Berkeley, CA 94720 United States
[4] Department of Agricultural and Resource Economics, University of California, Berkeley, Berkeley, California 94720, United States
[5] National Bureau of Economic Research, Cambridge, Massachusetts 02138, United States
[6] Life Cycle, Economics & Agronomy Division, Joint BioEnergy Institute, Emeryville, CA 94608 United States
[7] Biological Systems & Engineering Division, Lawrence Berkeley, National Laboratory, Berkeley, CA 94720 United States

*Co-corresponding authors: auffhammer@berkeley.edu, cdscown@lbl.gov

Pages S1- S59

Tables S1-S17

Figures S1-S14

Procedure S1: Peaker Replacement Behavior and Sizing Optimization

The following optimization formulation is used to determine the charging behavior and rated power of the BESS when operating to offset natural gas combustion at peaker plants. The formulations represent the optimization for a single 24-hour period, repeated for everyday from 2018 through 2020. The maximum rated power observed over the entire three-year span is set as the rated power for the BESS.

(1) $$\min(R * P_{Batt} + \sum_i^n P_{Elec,i} * r_i)$$

Such that:

(2) $$0 \leq r_i \leq R \leq R_{Cap}$$

(3) $$z_i = z_{i-1} + \eta * r_{i-1} - \frac{Q_{i-1}}{\eta}$$

(4) $$0 \leq z_i \leq R * D$$

(5) $$z_0 = z_p$$

(6) $$r_i * Q_i = 0$$

(7) $$R \geq Q_i$$

Table S1 defines the variables and scripts. For the first optimization period observed from 2018 through 2020, z_p is set to zero. Negative prices are artificially set to near-zero positive values during optimization in order to avoid unrealistic charging behavior – when calculating costs and revenues, negative prices are allowed. 24 hour segments are optimized at a time to mimic the day-ahead market structure. When optimization is infeasible for 24 hour periods (generally if discharging events occur too near the beginning of these periods and/or if the discharge event is very large), the period is expanded earlier by an additional 24 hours until feasible. The largest R solved from the optimization of the all the periods in the three-year span considered is set as the rated power of the BESS. Our methods ensure that R remains less than r_{cap} (the rated power of the existing peaker plant) for all optimization periods to minimize impacts associated with upgrading transmission and distribution infrastructure while still meeting the historic output of the peaker plant, Q_i. The price for increasing energy storage is set artificially high to minimize battery size while simultaneously minimizing the cost of purchasing electricity while still requiring that the BESS have sufficient energy storage capacity to displace all considered discharge periods from purchased electricity. The largest energy capacity from all 24 hour segments optimized and the annual number of cycles, are used to size the BESS such that it maintains its rated energy capacity despite calendar and cycling capacity fade.

Table S1. Natural Gas Peaker Replacement Optimization Definitions

Variable/Script	Definition
i	Hour timestep
n	Maximum hour observed, set to 24
p	Final timestep from prior optimization period
R	Required rated power (kW) for the optimization period
P_{Batt}	BESS price per kW, set artificially high to dissuade increasing rated power
$P_{Elec, i}$	Historical electricity price per kW from 2018 through 2020
r_i	BESS charging in kW
R_{Cap}	Maximum allowed charging per period in kW, set to the rated power of the peaker plant
z_i	State of charge of the BESS in kWh
η	One way efficiency of the BESS, set to square root of 0.85
Q_i	Fixed BESS discharging in kW from historical peaker output from 2018 through 2020
D	Duration of BESS, set to 4

Variables and scripts used in the optimization along with their associated definitions.

Procedure S2: Additional Peaker Replacement Characteristics

The initial optimization indicated that a BESS must have a large rated power and energy capacity to fully offset a natural gas peaker plant. In order to reduce the potential sizing and CapEx of a BESS, we performed an analysis in which the top n^{th} percentile load events from the historical activity of a natural gas peaker plant are excluded from the optimization above. Figure S1a, S1b, and S1c illustrate the results of the performed analysis. Figure S1a plots the required rated power of the BESS against the percentile of load events excluded. Figure S1b plots the annual load passing through the BESS against the percentile of load events excluded. Figure S1c plots the annual load passing through the BESS against the normalized required rated power of the BESS.

Figure S1a illustrates how excluding the top 5th percentile of load events from the optimization allows for a significantly reduced rated power for all BESS explored, in some cases even reducing the required rated power by around 80%. In Figure S1b, the impact of excluding the top n^{th} percentile of load events on the annual load passing through the BESS can be observed. In Figure S1c, the effect of reducing the required rated power of a BESS on the annual load passing through the system can be observed. Significant size reductions can be achieved by excluding the 5th percentile of load events while still meeting over 75% of the total annual load that would otherwise be offset by a fully sized BESS. Therefore, all BESS are optimized to meet the 95th percentile load event for natural gas peaker replacement – a decision that will significantly reduce CapEx and increase the feasibility of BESS for natural gas peaker replacement.

Figure S1: Relationship Between Percentile of Load Events Excluded, BESS Rated Power, and Offset Annual Load by BESS.

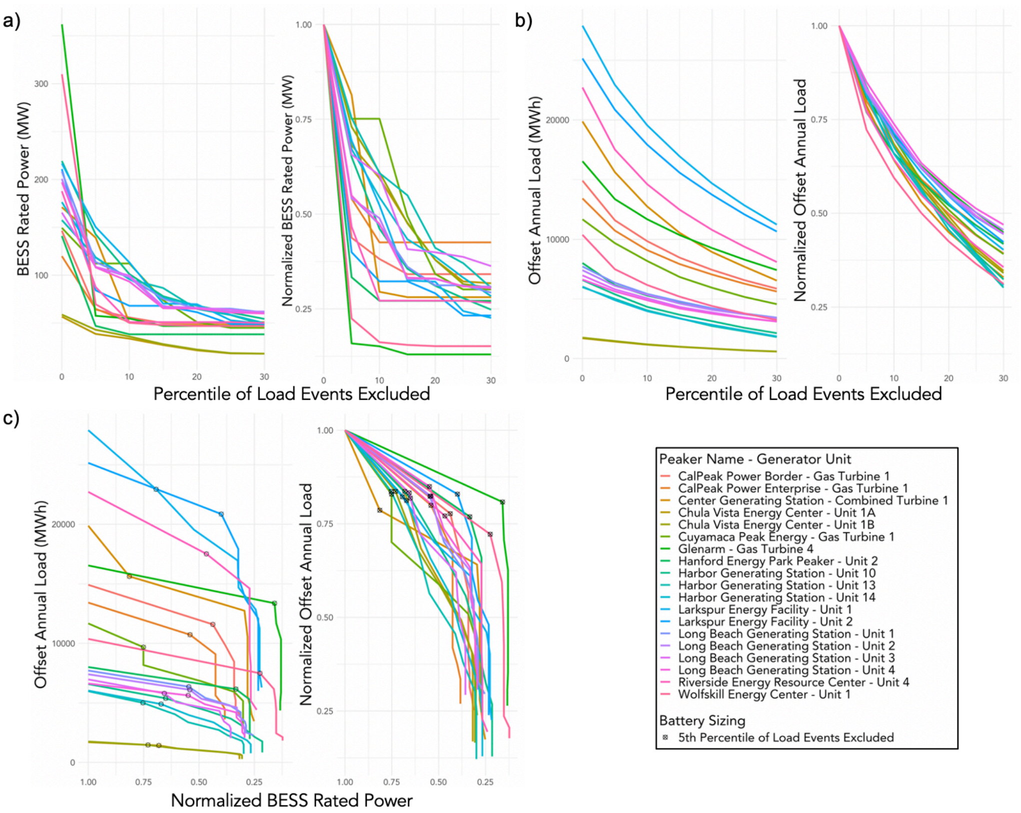

Each color represents the characteristics of each peaker studied and each line represents an individual BESS offsetting peaker plant activity. The “Percentile of Load Events Excluded” refers to the omission of discharge events by the events’ sizes. For example, at the 5th percentile of load events excluded, any discharge event from 2018 to 2020 that is in the top 5th percentile by size (MWh) has been omitted from the study. As a result, the required rated power of the BESS to offset all considered peaker activity is reduced, since less storage capacity is required. (A) The plot visualizes the relationship between the required BESS rated power and the percentile of load events excluded when sizing through optimization. (B) The plot visualizes the relationship between the offset annual load by the BESS and the percentile of load events excluded when sizing through optimization. (C) The plot visualizes the relationship between the offset annual load by the BESS and the BESS rated power, with an additional point indicating where the BESS are sized for this study, excluding the 5th percentile of load events.

Procedure S3: Arbitrage Behavior Optimization

The following optimization formulation is used to determine the charging and discharging behavior of the BESS when performing arbitrage. The formulations represent the optimization for a single 24 hour period, repeated for everyday from 2018 through 2020.

$$\min \sum_{i}^{n}\{(Q_i - r_i) * P_{Elec,i} - \frac{Q_i}{R*D} * P_{Cycle}\} \tag{8}$$

Such that:

$$0 \leq r_i \leq R \leq R_{Cap} \tag{9}$$

$$z_i = z_{i-1} + \eta * r_{i-1} - \frac{Q_{i-1}}{\eta} \tag{10}$$

$$0 \leq z_i \leq R * D \tag{11}$$

$$z_0 = z_p \tag{12}$$

$$r_i * Q_i = 0 \tag{13}$$

For the first optimization period observed from 2018 through 2020, z_p is set to zero. Negative prices are artificially set to near-zero positive values. When optimization is infeasible for 24 hour periods, the period is expanded early by an additional 24 hours until feasible. The optimization skips periods when the BESS is participating in natural gas peaker replacement. A cost penalty equivalent to the fractional cost of the battery degraded from cycling (Table S15) is incurred with discharging. The price of electricity controls the revenues from selling electricity and the costs of purchasing electricity. Table S2 outlines the variables used for modeling.

In order to perform the optimization on a convex set, we artificially set the discharging event of maximum power at the hour with the highest electricity price during the period. If arbitrage is found to be profitable, we set a second maximum discharge event at the hour with the next highest hour and perform the optimization again. We repeat this until profits decrease; after that we use the prior discharge scenario. If we find the initial optimization scenario unprofitable, then no arbitrage occurs over the period.

After the number of cycles associated with arbitrage is determined, the upfront size of the BESS is increased to ensure it can maintain its rated power despite the additional degradation from cycling for arbitrage.

Table S2: Natural gas peaker replacement optimization definitions.

Variable/Script	Definition
i	Hour timestep
n	Maximum hour observed, set to 24
p	Final timestep from prior optimization period
R	Required rated power (kW) for the optimization period, set to results from peaker replacement optimization
$P_{Elec,\ i}$	Historical electricity price per kW from 2018 through 2020
P_{Cycle}	Incurred cost per cycle attributable to additional upfront sizing to compensate for degradation from battery cycling. See Table S12 for description of P_{Cycle} as it varies by battery chemistry.
r_i	BESS charging in kW
R_{Cap}	Maximum allowed charging per period in kW, set to the rated power of the peaker plant
z_i	State of charge of the BESS in kWh
η	One way efficiency of the BESS, set to square root of 0.85
Q_i	BESS discharging in kW, set iteratively
D	Duration of BESS, set to 4

Variables and scripts used in the optimization along with their associated definitions.

Procedure S4: Frequency Regulation and Mileage Revenue Modeling

The following modeling approach is modified from the description of frequency regulation markets in Xu 2016[1] and is used to estimate revenues from participation in frequency regulation and mileage. Generally, total revenue can be broken into three main components, Capacity, Mileage, and Fast Response:

$$Total\ Revenue = Capacity\ Rev. + Mileage\ Rev. + Fast\ Response\ Rev.$$

Capacity revenue represents the revenue earned from bidding a capacity available for charging or discharging (regulation up or regulation down). It takes the following form:

$$Capacity\ Rev. = \sum_{i=1}^{n} Ci * P_{Ci}$$

Where C_i represents the bid capacity (kW) for each hour i, and P_{Ci} represents the clearing price ($/kW) for capacity for each hour i.

Mileage revenue represents the revenue earned from charging or discharging a portion of a system's bid capacity and takes the following form:

$$Mileage\ Rev. = \sum_{i=1}^{n} Ci * M_i * P_{Mi} * \ \rho_i$$

Where C_i represents the bid capacity (kW) for each hour i, M_i represents the proportion of that capacity that is actually called upon for regulation up or down, P_{Mi} represents the clearing price ($/kW) for mileage for each hour i, and ρi represents an accuracy score from 0 to 1 that varies based on the performance of the system.

Fast response revenue is an additional stream of revenue that is employed by some independent system operators but is not used by CAISO. Thus, it is excluded from this study. More information on this revenue stream and all other revenue components can be found with Xu 2016.[1] Additionally, environmental impacts associated with frequency response are not included in NPV results due to their minor contributions and complexity of modeling relative to other emission categories.

Procedure S5: BESS CapEx Modeling

The CapEx of the BESS can be categorized into 8 primary components: the battery, the inverter, the container balance of systems (BOS), the electrical BOS, the structural BOS, installation and labor sales tax, and developer costs. These primary cost components align with categories specified by NREL in past works[2,3] with some modifications to allow for a greater level of granularity inspired by PNNL modeling.[4] These primary components are made up of secondary components that generally represent a single type of equipment, material, or soft cost. Table S3 shows the 8 primary cost components and their associated secondary components.

The battery primary component consists of the Li-ion battery module itself and the systems contained within it. This generally includes the Li-ion cells, miscellaneous electronics associated with module level controls, and housing/cooling elements. We determine the sizing of this component by the required rated power determined from optimization for natural gas peaker replacement, the amount of annual cycles determined from optimization for natural gas peaker replacement and arbitrage, and the scenario specific system lifetime and battery replacement timeframe. We provide additional details on the sizing of the battery system and all other primary components in the Procedures S8 and S9.

Table S3: Primary and secondary components of BESS.

Primary Component	Secondary Component
Battery System	Battery Module
Inverter	Inverter
Container BOS	Thermal Regulation, Fire Suppression, Gas/Fire Detection
Electrical BOS	Transformer, Switchgear, Substation*, Conductors, Conduits, Communications
Structural BOS	Battery Housing, Battery Racks, Inverter Housing, Foundations
Installation and Labor	Battery Module Installation, Inverter Installation, Electrical BOS Installation, Structural BOS Installation, Site and Misc. Labor
Sales Tax	State Taxes, Local Taxes
Developer Costs	Developer Overhead, EPC Overhead, Permitting, Inspection, Interconnection, Contingency, Net Profit, Environmental Study and Mitigation, Land Acquisition

* Substation only included if certain model conditions met, i.e. rated power exceeds 100 MW[2–4]

Procedure S6: Life-Cycle Assessment

A hybrid process-based/physical units-based input-output method was used to calculate the life-cycle GHG footprint of each BESS using an approach described in prior studies.[5–7] LCA generally considers four phases of a product's lifecycle: material extraction and processing, assembly, use, and end-of-life. For a BESS and its subcomponents, values pertaining to the impacts associated with material extraction and processing are generally sourced from GREET2 as well as other bodies of literature or first order estimations, all of which are presented in Table S13. For the assembly of BESS and its subcomponents, distributions and ranges are assembled from literature values to best reflect the many possibilities in cell manufacturing. These distributions are presented in Table S11. The local environmental damages associated with Li-ion cell manufacturing are excluded due to the unavailability of data on foreign energy mixes, generation dynamics, and on site fuel consumption. However, as illustrated in Figure 4, environmental damages are minor relative to monetary considerations, and the exclusion of these damages will likely not significantly alter the final NPV. Use phase impacts are determined through the analyses Experimental Procedure previously outlined for each potential revenue stream. Additionally, the impacts associated with induced and offset electricity demand are outlined in the following section. A BESS is assumed to be landfilled at its end-of-life, with the partial recovery of certain high value materials like the HVAC refrigerant and fire suppressant. No battery recycling is assumed due to the current, limited state of the battery recycling supply chain.[8,9] Impacts associated with material transportation and onsite BESS construction are considered negligible, excluding the emissions associated with concrete. Future extraction and processing, manufacturing, and end-of-life conditions are assumed to mimic current conditions. Additionally material breakdowns and assumptions are presented in Table S13 and S14.

Procedure S7: Induced and Offset Electricity Demand

In order to understand the impacts of battery charging and discharging behavior – we must know something about the electricity and emissions associated with charging a battery and the electricity that the battery offsets. We use a regression-based reduced form approach to this problem based on widely used Experimental Procedure in the economics literature.[10–12] We build a model to predict individual plant behavior based on the aggregate demand for electricity in a plant's region. This model allows us to statistically estimate which plants respond to the increased electricity load from battery charging.

We conducted this analysis by leveraging EPA's Continuous Emissions Monitoring Systems (CEMS) data—which allows us to observe hour-by-hour power output and emissions for nearly all of the plants in the Western Interconnection. The EPA reports CEMS data for each generating unit within a plant separately. The data we use consists of hourly measures of generation in MWh, CO_2, SO_2, NO_X and $PM_{2.5}$ for 574 unique generating units contained within 224 power plants. CEMS data are highly accurate given the requirement of these data for regulatory purposes and because the methodology used to impute missing data creates a disincentive for firms to skirt regulations by turning off their monitoring equipment.[13]

In order to relate emissions to system load, we merged these CEMS data with aggregate demand (or load) data for the relevant North American Electric Reliability (NERC) regions servicing California. Since California operates a fairly distinct wholesale market from the rest of the Western Interconnection, we tabulated loads via Federal Energy Regulatory Commission (FERC) Form 714 data for California and the remainder of WECC separately.

With these two datasets, we estimated a model of how each generating unit responds to additional electricity load, which allows us to measure the explicit impacts from increased demand for electricity derived from battery charging. Specifically, we ran a set of generator-specific regressions that relate a generator's power output and emissions to aggregate electricity demand for the two NERC regions separately. As such, the dependent variable in each of our regressions is an hourly measure of generator i's hourly generation or emissions (CO_2, SO_2, NO_X, PM_{25}). We regress each dependent variable on a set of variables constructed from the aggregate demand for each NERC region. We allow for heterogeneity in the responsiveness of generators by hour-of-day since it is likely a plant responds to increased demand at 5AM differently than 3PM. Functionally, we estimate the following equation to determine how plants respond to additional system load:

$$Y_{it} = \sum_{h=1}^{24} \sum_{n \in \{CA, West\}} \beta_{int} * HOUR_h LOAD_{jt} + \sum_{h=1}^{24} \sum_{m=1}^{36} \gamma_{ihm} HOUR_h MONTH_m + \varepsilon_{it}$$

Here Y_{it} represents hourly generation or emissions for generator i in hour t. The set of $\boldsymbol{\beta}$int coefficients measure the marginal effect of additional (or decreased) load from NERC region n in hour-of-day h on that generator's emissions or generation. Allowing these changes to vary by NERC region n, allows, for example, a generator in California to respond to increased electricity demand in California in a different manner than it responds to increased electricity load in

Nevada. This is an important feature of the market given that NERC regions are interconnected– but remain separate entities when it comes to market-based generation behavior. We also note the $\boldsymbol{\beta}_{int}$ coefficients are specific to generator i, allowing for these marginal effects to differ by generator. The set of γ_{ihm} coefficients act as high-dimensional fixed effects that net out any fixed shifts in behavior for generator i at the sample-month-by-hour-of-day level. These fixed effects soak up any seasonal or hour-of-day variation that may be related to trends in electricity demand. Hence, our $\boldsymbol{\beta}_{int}$ coefficients measure the impact of additional load net of any of this hour-of-day-by-sample month variation. We used these coefficients to calculate generator-specific power output and emissions responses to additional system load induced by battery charging in region n and hour h.

We next converted these changes in emissions into monetary damages. For CO_2, we simply multiplied each generator's emissions by the social cost of carbon since these damages do not vary across location. Air pollution damages (i.e. SO_2, NO_X and $PM_{2.5}$), however, vary depending on where they are emitted, so we use generator-specific marginal damage estimates from the EASIUR model to convert these damages into monetary units for each plant separately. The EASIUR model provides marginal damage estimates for 16,576 cells from a 148x112 grid representing the United States. For each generator, we determined the relevant grid cell based on the generator's GPS coordinates and apply marginal damage estimates from EASIUR to the induced emissions from charging for that generator. Since EASIUR reports monetized damages for three different emission altitudes (0M, 150M and 300M), we matched generators to the nearest altitude based on the generator or plant smokestack height. We report damages in 2020 USD throughout the paper.

Accounting for the impacts of offset generation from the peaker generator we replace with a battery storage facility presents an easier task. Since we model the battery storage facility to replace a given amount of observed generation for a specific generator, we can simply calculate the offset emissions associated with that replaced generation directly based on the observed hourly data in the EPA CEMS data. We do not replace all generation hours for each generator in our final model, so we only remove the emissions associated with the hours our model selects to offset with the battery storage facility. For hours where we only partially offset generation, we remove emissions in proportion to the ratio of offset generation and total generation for that hour. We convert these reductions in emissions to monetary units using the same procedure outlined above for induced emissions from charging.

Table S4: Connected or under contract large-scale battery energy storage systems in CA.[14]

Name	Location	Rated Power (MW)	Commissioned Date
Gateway BESS	San Diego, CA	50	August 2020
NextEra Blythe BESS	Riverside, CA	63	August 2021
Vistra Moss Landing BESS	Moss Landing, CA	400	August 2021
Coso BESS	Little Lake, CA	60	August 2021
Diablo BESS	Contra Costa County, CA	200	August 2021
Elkhorn BESS	Moss Landing, CA	182.5	April 2022
Beaumont BESS	Beaumont, CA	100	August 2023
Edwards Sanborn BESS	Mojave, CA	169	August 2023
Canyon Country BESS	Santa Clarita, CA	80	October 2023
MOSS350 BESS	Moss Landing, CA	350	August 2023
Inland Empire BESS	Rialto, CA	100	April 2024
Corby BESS	Vacaville, CA	125	June 2024
Kola BESS	Tracy, CA	275	June 2024
Nighthawk BESS	Poway, CA	300	June 2024
Caballero BESS	Nipomo, CA	100	June 2024

Procedure S8: Additional details on battery sizing.

The battery system of the Li-ion BESS is sized to ensure that the whole system can output its rated power and energy capacity over the entire lifespan of the battery, either 7.5 years or 10 years, before the battery is replaced. The following equations are used to perform this sizing.

(14) $$r_{Cal}\left[\frac{\%}{Cycle}\right] = \left(1 - EOL^{1/T_{Life}}\right) * \left(\frac{1}{Cycles\ Per\ Year}\right)$$

(15). $$r_{Cyc}\left[\frac{\%}{Cycle}\right] = \left(1 - EOL^{1/C_{Life}}\right)$$

(16) $$r_{Tot} = r_{Cal} + r_{Cyc}$$

(17) $$D_n = D_0 * \prod_{1}^{i=n}(1 + r_{Tot} * D_{i-1})$$

(18) $$R_{Adj} = \frac{R}{D_n * RTE}$$

Table S5: Battery sizing definition.

Variable/Script	Definition
r_{Cal}	Battery degradation attributable to calendar aging, converted to % per cycle
r_{Cyc}	Battery degradation attributable to cycling, converted to % per cycle
T_{Life}	Shelf life in years, varies by cathode chemistry, collected from literature. See Table S6
C_{Life}	Cycle life in number of cycles, varies by cathode chemistry, collected from literature. See Table S6
EOL	Battery state of health (%) at end of life. Set to uniform distribution of 70% to 80% to reflect uncertainty in reporting in literature
Cycles Per Year	Equivalent cycles per year from peaker replacement and arbitrage
r_{Tot}	Total battery degradation attributed to each cycle
D_n	Required initial depth of discharge
D_0	Maximum allowable depth of discharge, set to 95% [15]
R_{Adj}	Adjusted battery capacity to achieve rated power over entire battery lifespan
R	Rated power
RTE	Round trip efficiency calculated from battery efficiency, transformer efficiency, inverter efficiency, HVAC power draw, and miscellaneous power draw.

Table S5 defines variables used for sizing. This formulation does not mimic the real behavior of battery degradation but rather estimates the required battery capacity to maintain a rated power throughout its lifetime using high level properties. Additionally, other options exist for maintaining a rated power, but the oversizing approach has the least uncertainty with battery pricing and is frequently used for projects this size.[16,17]

Table S6: Battery shelf and cycle life definitions by chemistry.[18–26]

**Chem**	**T_{Life}**	**C_{Life}**
NCA	8 – 10	2000 – 3500
NMC	8 – 10	2000 – 3000
LFP	8 – 12	3000 – 5000

Procedure S9: Additional details on other primary component sizing.

The container BOS primary component consists of the controls and balance of systems that support battery modules. This includes equipment associated with thermal regulation (i.e. HVAC or cell cooling) , fire suppression, and gas detection.[27–30] Each containerized group of battery modules has its own container BOS.[4]

The inverter primary component consists of the bidirectional inverters used to convert the energy from AC to DC while the system is charging and from DC to AC while the system is discharging. In utility scale BESS, multiple bidirectional inverters may be used, each one connected to a single or set of containers housing battery modules.[16,27,28,31,32]

The electrical BOS primary component consists of the transformers, switchgear and breakers, conductors, conduits, and communications required to transfer energy within the system and onto the grid infrastructure. The quantity and properties of transformers in the system can vary by grid properties such as voltage and interconnection limits. The configuration of conductors and conduits is highly variable and dependent on-site constraints, system design, and grid interactions. Additional equipment may be required for site integration, such as a substation, depending on the size of the energy storage system and the characteristics of the grid.[27,28,30,32]

The structural BOS primary component includes the racks and housing for the batteries, the inverter housing, and foundations required for the equipment in the system.[27,28]

The installation and labor primary component is made up of the costs associated with the wages of workers and the time spent installing the equipment tied to each of the previous primary components, as well as any other miscellaneous work that must be performed. This miscellaneous work may include grading, trenching, and hauling among others.[27] [28]

Developer costs are generally defined as the soft costs that are passed onto the project developer and include developer overhead, permitting, inspection, interconnection, contingency, net profit, environmental study, and environmental mitigation. Developer overhead is defined as the fees required for the developers to maintain their operations. EPC overhead is defined as the fees required for the EPC company to maintain their operations. Permitting is defined as the administrative review and permits required to construct a battery energy storage project. Interconnection is defined as all the grid upgrades required to integrate the battery energy storage system to the grid. Inspection is defined as the testing that must occur before the BESS is fully integrated into the grid. Contingency is defined as a factor to account for any unforeseen events that may occur during development of the BESS. The environmental study is defined as the study that must be performed to determine if further environmental mitigation is required, while environmental mitigation is defined as the additional site work that may be required to minimize damage to impacted ecosystems in our surrounding the site.[16,17,27,28,33]

Sales tax consists of both local and state taxes that are applied as a factor to the total cost of materials required.[34]

These primary components and the secondary components they include are visualized in Figure S2.

Figure S2: Primary components, secondary components, and interactions of a BESS.[4,27]

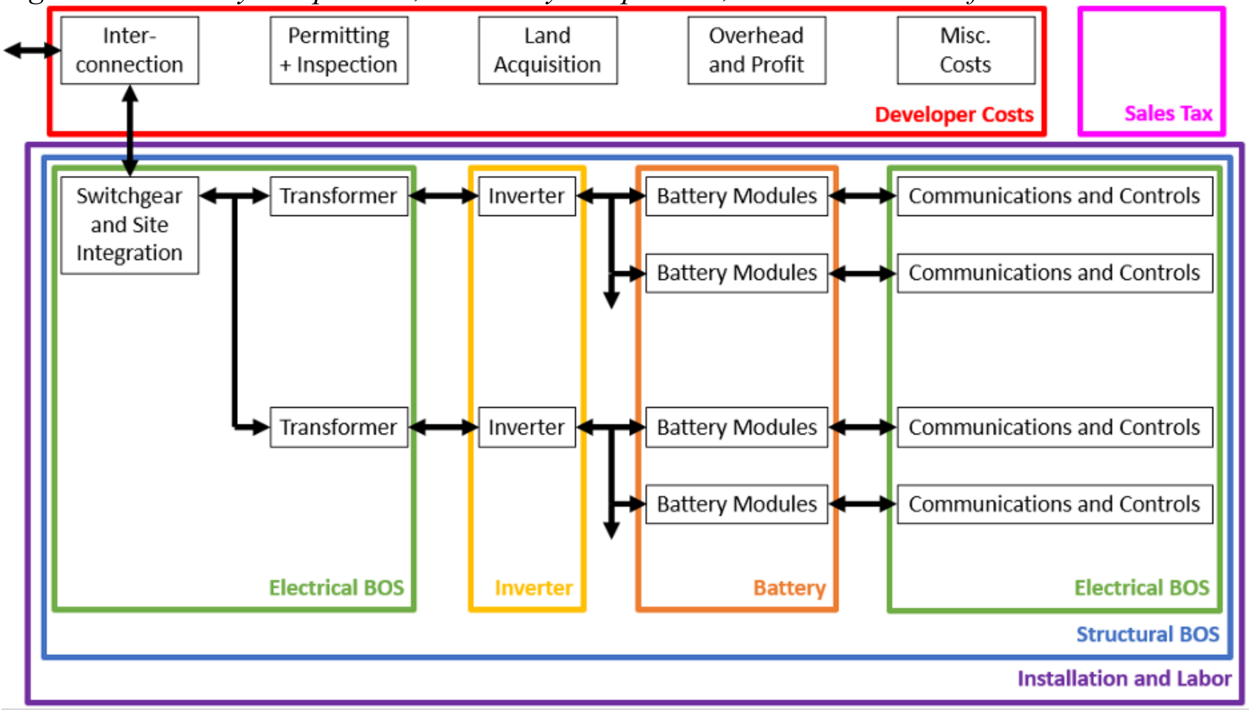

Schematic representation showing the secondary components included in each primary component and their interactions of the BESS when discussing CapEx.

Table S7: Characteristics of natural gas peaker plants in CA and optimized characteristics of BESS.

Replaced Peaker Plant	CAISO Region	Peaker Rated Power (MW)	Opt. BESS Rated Power (MW)	Annual Eq. Cycles - Peaker Rep.	Annual Eq. Cycles - LFP Arbitrage	Annual Eq. Cycles - NCA Arbitrage	Annual Eq. Cycles - NMC Arbitrage
Long Beach Generating Station - Unit 1	SP15	65	113.72	13.89	9.62	4.43	1.19
Long Beach Generating Station - Unit 2	SP15	66	108.67	13.94	9.82	4.30	0.86
Long Beach Generating Station - Unit 3	SP15	66	108.62	13.24	9.67	4.20	0.86
Long Beach Generating Station - Unit 4	SP15	65	108.11	12.92	10.27	4.66	1.00
Harbor Generating Station - Unit 10	SP15	47	142.65	9.32	7.47	4.20	1.92
Harbor Generating Station - Unit 13	SP15	47	118.69	10.48	8.98	4.88	1.95
Harbor Generating Station - Unit 14	SP15	47	118.37	10.20	8.87	4.90	2.28
Glenarm - Gas Turbine 4	SP15	47	57.55	58.07	7.69	3.95	1.29
CalPeak Power Border - Gas Turbine 1	SP15	51	64.06	45.24	9.75	5.31	2.39

Cuyamaca Peak Energy - Gas Turbine 1	SP15	45	112.24	21.51	6.62	3.57	1.47
CalPeak Power Enterprise - Gas Turbine 1	SP15	51	64.81	41.38	9.05	5.25	3.02
Chula Vista Energy Center - Unit 1A	SP15	18	38.62	9.08	11.53	6.64	3.22
Chula Vista Energy Center - Unit 1B	SP15	18	42.98	8.42	10.51	5.97	2.90
Larkspur Energy Facility - Unit 1	SP15	49	150.29	38.16	3.56	1.85	0.76
Larkspur Energy Facility - Unit 2	SP15	49	84.20	61.94	7.37	4.22	2.04
Hanford Energy Park Peaker - Unit 2	NP15	47	47.00	32.77	7.33	4.42	2.92
Wolfskill Energy Center - Unit 1	NP15	48	69.60	26.91	6.90	4.31	2.76
Riverside Energy Resource Center - Unit 4	SP15	51	87.64	49.98	7.86	5.24	3.15
Center Generating Station - Combined Turbine 1	SP15	48	139.21	28.07	4.52	2.20	0.91

Figure S3: Example State-of-Charge for BESS Offsetting Long Beach Generator Unit 1

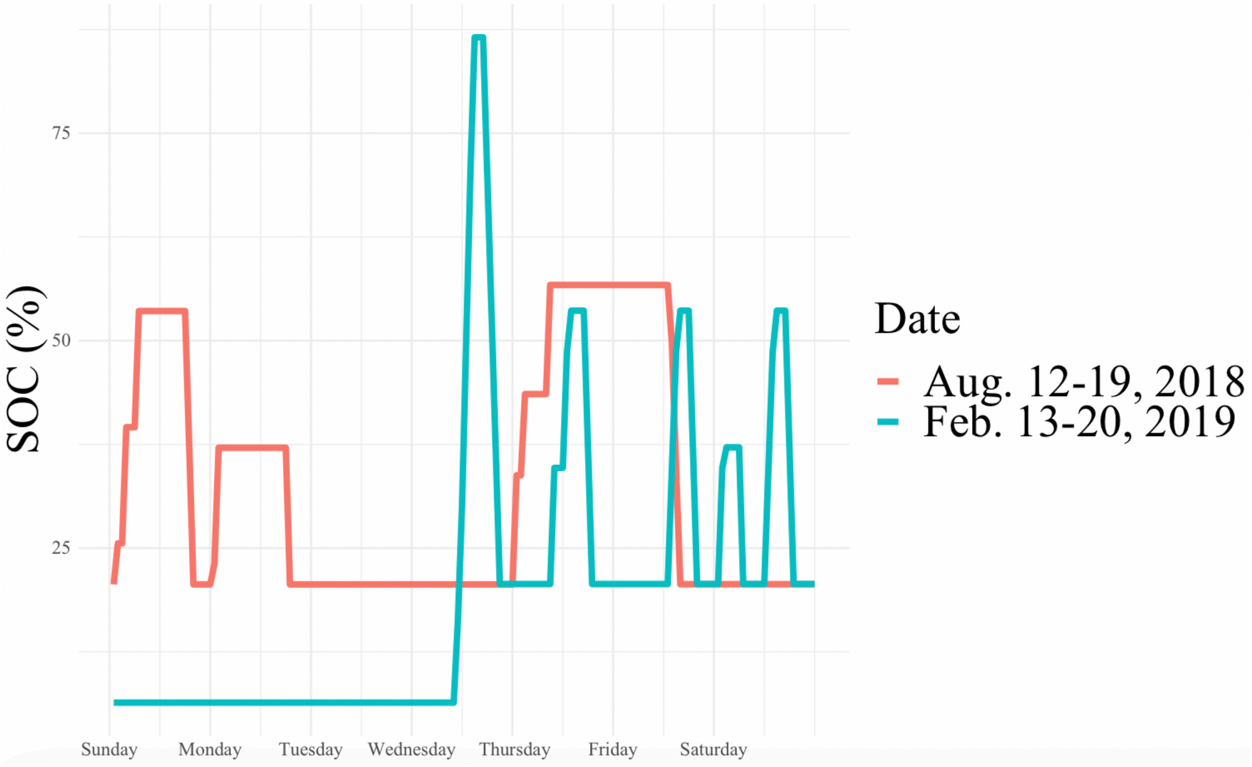

Figure S4: Example CapEx of BESS

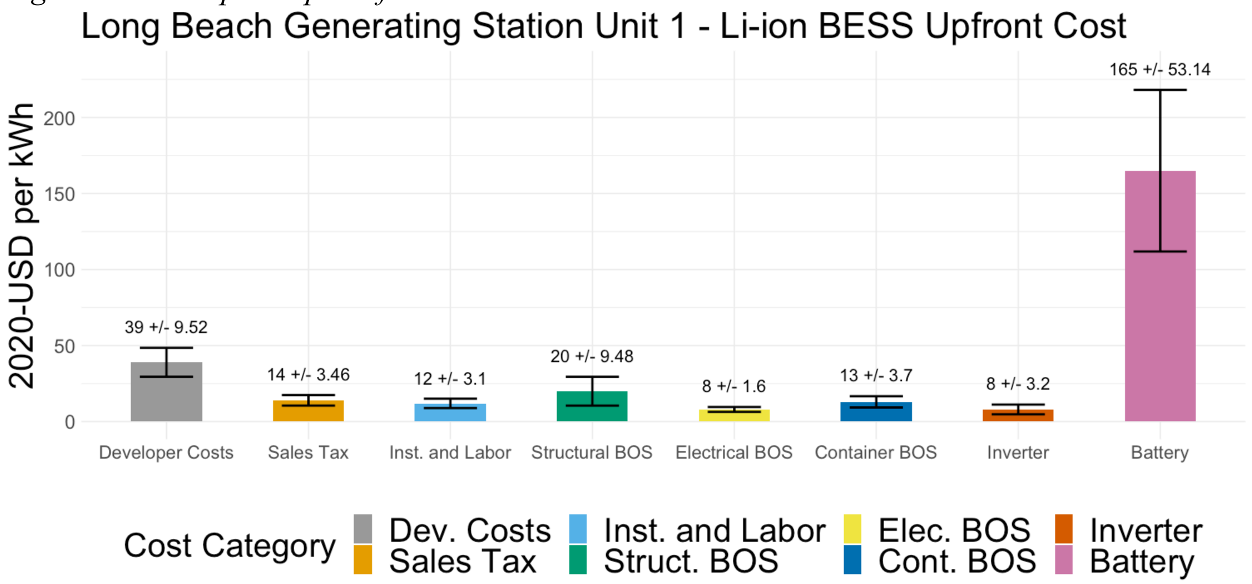

The CapEx for the BESS replacing the Long Beach Generating Station Unit 1 peaker plant is presented by category. An LFP cathode chemistry is assumed, with a 15 year lifespan and battery replacement at 7.5 years. Error bars represent two standard deviations with 500 runs in the Monte Carlo analysis.

Table S8: Distributions and values for design and cost parameters of BESS.

Component/Parameter	Distribution/equation/description	Source
LFP Module	Price ($/kWh): lognormal distribution; alpha = 99, beta = 1.25, mu = 0, sd = 5, min = 99, max = 225<br><br>Additional rack multiplier of 1.07 on price to account for additional structural components due to reduced energy density<br><br>Labor: labor hours = 1 hr/module; labor rate = 50.70 $/hr<br><br>One way efficiency: Uniform distribution; min = 98%, max = 99%	[16,28,35–37]
NCA Module	Price ($/kWh): lognormal distribution; alpha = 115, beta = 5*10^-6, mu = 0, sd = 20, min = 115, max = 415<br><br>Labor: labor hours = 1 hr/module; labor rate = 50.70 $/hr<br><br>One way efficiency: Uniform distribution; min = 98%, max = 99%	[16,28,35,37]
NMC Module	Price ($/kWh): lognormal distribution; alpha = 115, beta = 100, mu = 0, sd = 5, min = 115, max = 525<br><br>Labor: labor hours = 1 hr/module; labor rate = 50.70 $/hr<br><br>One way efficiency: Uniform distribution; min = 98%, max = 99%	[16,28,35,37]
Inverter	Price ($/W): Triangular distribution; min = 0.04. mode = 0.06, max = 0.08<br><br>Labor: modeled as transformer<br><br>Losses: Normal distribution; mean = 1.83%, sd = 0.599%	[16,28,36,38]<br><br>Sampled from available products

Transformer	Price ($): Mode interpolated by size, see Table S10. Triangular distribution; max/min = mode +/- 12.5%*mode<br><br>Labor: Labor hours interpolated by size, see Table S10; labor rate = $60.07/hr<br><br>Losses: Normal distribution; mean = 0.47 %, sd = 12.5% * mean	[16,28,39]
Switchgear	Price ($/MW): Triangular distribution; If under electrical BOS size is less than 25 MW, mode = 100,000 USD. If electrical BOS is greater than 25 MW, mode = 100,000 USD + 1,333.33 $/MW. max/min = mode +/- 12.5%*mode<br><br>Labor: modeled as transformer	[16,28]
Interconnection	Price ($/MW): Uniform distribution; min = 1,000,000 USD, max = 3,000,000 USD	[16,17,36]
Conductors	Price ($/ft): Uniform distribution: min = 2.5, max = 7.5<br><br>Labor: labor hours = 5.5 hrs per 100 ft; labor rate = $54.24/hr	[16,28,36]
Conduits	Price ($/ft): Normal distribution; mean = 18.9, sd = 3.4<br><br>Labor: labor hours = 0.16 hrs per foot; labor rate = $54.24/hr	[16,28]
BMS	Price ($): Uniform distribution; min = 200,000, max = 600,000<br><br>Labor: labor hours = 13.33 hrs/MW_rated_power; labor rate = $54.20/hr	[16,17,28,36]

Fire Suppression	<u>Price ($/Battery container):</u> Normal distribution; mean = 1975 + 1.76 * container_volume_ft^3, sd = 12.5% * mode<br><br><u>Labor:</u> labor hours = (2 + 0.1 * container_length_ft + containter_length/30) hours per container ; labor rate = $52.12/hr	[28–30,32]
HVAC	<u>Price ($/Battery container):</u> Uniform distributuion; min = $5,000/battery_container, max = $15,000/battery_container<br><br>Labor: labor hours = 6.67 hours per MW_rated_power,<br>Labor rate = $53.31/hr<br><br><u>Losses (%):</u> min = 0.5%, max = 2.5%	[28,36,40–42]
Battery Housing	<u>Price ($/container):</u> Normal distribution; mean is variable by container size (30,000 for 40 ft container, 15,000 for 20 ft container, 5,000 for cabinet), sd = 25% * mean<br><br><u>Labor:</u> modeled as transformer	[16,28,36]
Inverter Housing	<u>Price ($/container):</u> Normal distribution; mean is variable by container size (15,000 for 20 ft container, 5,000 for cabinet, 0 for in battery), sd = 25% * mean<br><br><u>Labor:</u> Installed with inverter	[16,28]<br><br>Sampled from available products
Foundation	<u>Price ($/cubic yard):</u> normal distribution; mean = $140/cubic yard, sd = 12.5% * mean<br><br><u>Labor:</u> labor hours = 0.957 hrs/cubic yard, labor rate = 46.00	[28]
Grading	<u>Labor:</u> labor hours and rates variable by site size, see Table S11	[28]
Trenching	<u>Labor:</u> Labor hours = 4 hrs/cubic_yard, labor rate = $46/hr	[28]
Backfill	<u>Labor:</u> Labor hours = 0.691 hrs/cubic yard, labor rate = $50.69/hr	[28]

Communications	Price ($): Uniform distribution; min = 100,000 USD, max = 300,000 USD<br><br>Labor: labor hours modeled as transformer, labor rate = $54.24/hr	[16,28,36]
Gas Detection Probes	Price ($/Battery Container): 630<br><br>Labor: Labor hours = 1 hr/battery_container, labor rates = $54.24/hr	[16,28,30]
Gas Detection Controllers	Price ($/Battery Container): 2925<br><br>Labor: Labor hours = 1 hr/battery_container, labor rates = $54.24/hr	[16,28,30]
Fire Detection	Price ($/Battery Container): 51.50<br><br>Labor: Labor hours = 1 hr/battery_container, labor rates = $54.24/hr	[16,28,30]
Misc. Power Loss	Losses (%): Uniform distribution; min = 1%, max = 5%	[16]
Total Labor	Normal distribution; mean = total labor cost, sd = 12.5% * mean	Assumed
EPC Overhead	25% total labor cost + 8.67% material cost	[16,27,28,36]
Sales Tax	National average = 6.5696%	[34]
Dev Overhead	(12% +.0315%/MW_rated_power) * (Total labor cost + material cost)	[27,28,36]
Permits	Varies by state, national set to 0	[16,27,36]
Inspection	Uniform distribution; min = 5,000 USD, max = 15,000 USD	[16,27,36]
Contingency	Uniform distribution; min = 0.03% * (Total labor cost + material cost), max = 0.05% * (Total labor cost + material cost)	[16,27,28,36]
Net profit	Normal distribution; mean = 5% * (Total labor cost + material cost), sd = 12.5% * mean	[16,27,28,36]

Environmental Study	Uniform distribution; min = 5,000 USD, max = 15,000 USD	[16]
Environmental Mitigation	Uniform distribution; min = 0 USD, max = 100,000 USD	[16]
Land	Uniform distribution; min = 125,000 USD, max = 375,000 USD	[16]

Table S9: Annual Output and Omitted Load by Peaker Sized for the 95th Percentile Load Event

Replaced Peaker	Rated Power (MW)	Avg. Annual Peaker Replacement Output (MWh)	Avg. Annual Omitted Load (MWh)
Long Beach Generating Station - Unit 1	114	6,360	1,360
Long Beach Generating Station - Unit 2	109	6,100	1,300
Long Beach Generating Station - Unit 3	109	5,810	1,170
Long Beach Generating Station - Unit 4	108	5,630	1,000
Harbor Generating Station - Unit 10	143	5,390	1,200
Harbor Generating Station - Unit 13	119	5,000	980
Harbor Generating Station - Unit 14	118	4,900	1,130
Glenarm - Gas Turbine 4	58	13,370	3,170
CalPeak Power Border - Gas Turbine 1	64	11,590	3,330
Cuyamaca Peak Energy - Gas Turbine 1	112	9,680	1,990
CalPeak Power Enterprise - Gas Turbine 1	65	10,730	2,700
Chula Vista Energy Center - Unit 1A	39	1,430	280
Chula Vista Energy Center - Unit 1B	43	1,470	290
Larkspur Energy Facility - Unit 1	150	22,940	4,960
Larkspur Energy Facility - Unit 2	84	20,860	4,290
Hanford Energy Park Peaker - Unit 2	47	6,160	1,850
Wolfskill Energy Center - Unit 1	70	7,490	2,880

Riverside Energy Resource Center - Unit 4	88	17,520	5,200
Center Generating Station - Combined Turbine 1	139	15,630	4,250

Table S10: Transformer price and labor hours by size.[28]

Size (MW)	Labor Hours	Price (USD)
0.15	30.769	9750
0.3	44.444	13900
0.05	50	19700
0.75	52.632	25000
1	76.923	29600
1.5	86.957	35200
2	100	44400
3.75	125	83500

Table S11: Grading labor hours and rates.[28]

Size Area (SF)	Labor hours	Labor Rate ($/hrs)
0 - 400	12	37.76
400 - 1,000	24	37.76
1,000 – 3,000	16	47.46
3,000 – 5,000	24	47.46
5,000 – 8,000	40	43.33
8,000 – 10,000	12	81.85
10,000 – 20,000	9	81.12
20,000 – 25,000	11.5	81.12
25,000 – 30,000	13.33	81.12
30,000 – 35,000	16	81.12
35,000 – 40,000	18	81.12
40,000 – 45,000	20	81.12
45,000 – 50,000	22	81.12
50,000 – 75,000	32	81.12
75,000 – 100,000	44	81.12

Table S12: Frequency regulation and arbitrage profit comparison.[43]

Peaker Plant	Instances where FR profits exceed Arbitrage profits	Potential Annual FR Profits Change	Potential Annual Arbitrage Profits Change
Long Beach Generating Station - Unit 1	99 of 128	$589,547	-$255,285
Long Beach Generating Station - Unit 2	101 of 128	$618,180	-$265,720
Long Beach Generating Station - Unit 3	101 of 128	$611,610	-$261,342
Long Beach Generating Station - Unit 4	103 of 133	$620,101	-$269,646
Harbor Generating Station - Unit 10	95 of 149	$421,873	-$186,109
Harbor Generating Station - Unit 13	101 of 155	$455,235	-$201,492
Harbor Generating Station - Unit 14	96 of 152	$439,535	-$193,935
Glenarm - Gas Turbine 4	52 of 70	$206,180	-$99,168
CalPeak Power Border - Gas Turbine 1	55 of 85	$290,338	-$116,934
Cuyamaca Peak Energy - Gas Turbine 1	76 of 114	$302,456	-$133,897
CalPeak Power Enterprise - Gas Turbine 1	51 of 79	$285,913	-$113,591
Chula Vista Energy Center - Unit 1A	108 of 164	$179,922	-$81,687

Chula Vista Energy Center - Unit 1B	109 of 165	$181,276	-$82,188
Larkspur Energy Facility - Unit 1	53 of 76	$241,310	-$105,043
Larkspur Energy Facility - Unit 2	57 of 84	$251,139	-$115,036
Hanford Energy Park Peaker - Unit 2	44 of 61	$228,193	-$77,197
Wolfskill Energy Center - Unit 1	52 of 73	$246,754	-$83,121
Riverside Energy Resource Center - Unit 4	46 of 71	$247,419	-$111,019
Center Generating Station - Combined Turbine 1	70 of 90	$310,380	-$140,728

Instances in 2018 to 2020 where frequency regulation (FR) activity profits exceed arbitrage profits in the same time period, as well as the potential annual change in FR and arbitrage profits from these instances; determined from CAISO AS clearing prices and CAISO hour ahead markets.[43]

Table S13. Monthly energy for frequency regulation markets and monthly energy discharged and charged by batteries in CAISO.[43,44]

Month	Regulation Up (MWh)	Regulation Down (MWh)	Battery Discharge (MWh)	Battery Charge (MWh)
January 2022	368,820	538,900	102,520	140,010
February 2022	309,070	468,660	101,090	166,390
March 2022	338,650	558,430	76,270	155,490
April 2022	325,630	580,410	88,280	188,050
May 2022	312,240	551,580	94,140	210,510
June 2022	254,840	552,790	159,390	238,720

Figure S5: Net present values of BESS with LFP cathode chemistry and a social cost of carbon of \$51/tonne CO_{2eq}.

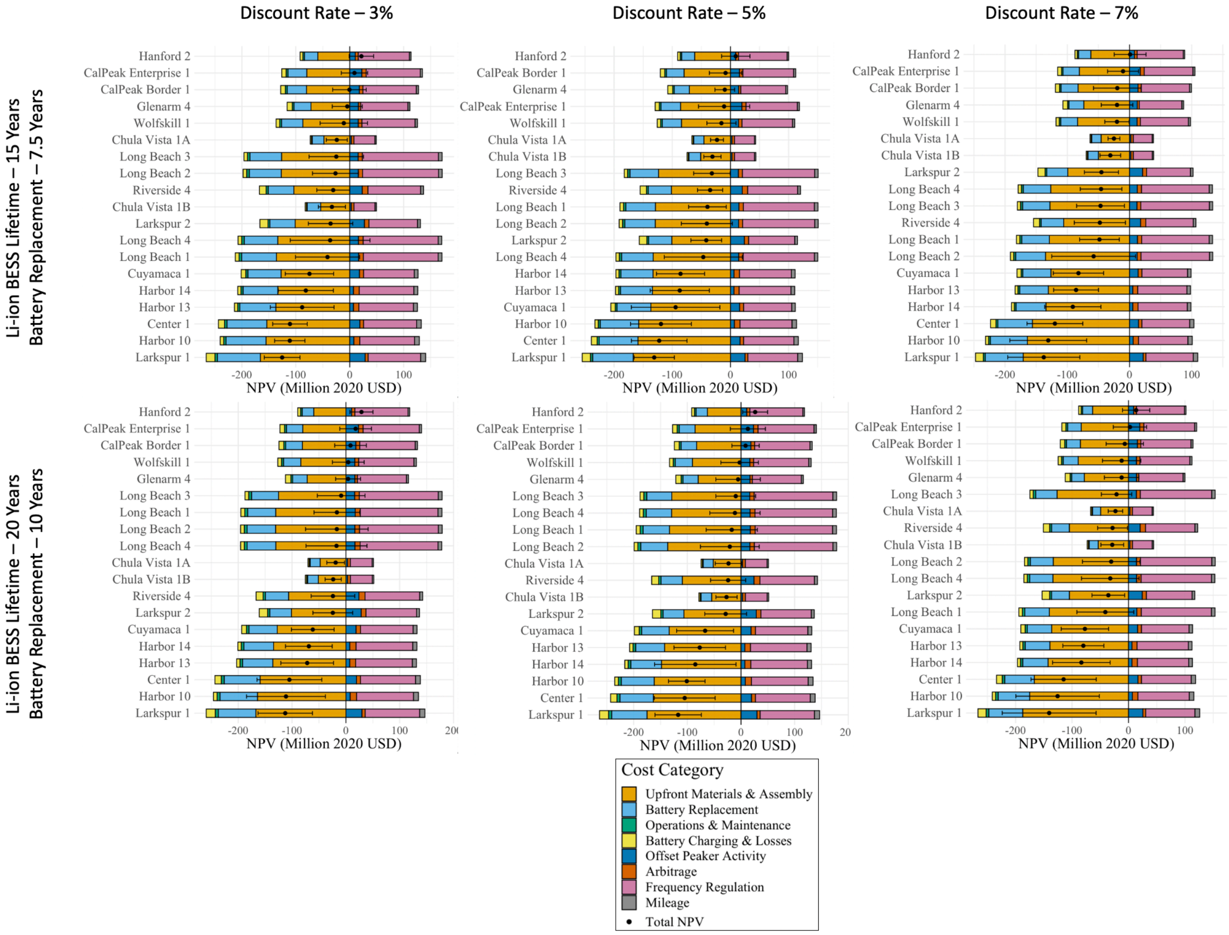

Figure S6: Net present values of BESS with LFP cathode chemistry and a social cost of carbon of \$185/tonne CO_{2eq}.

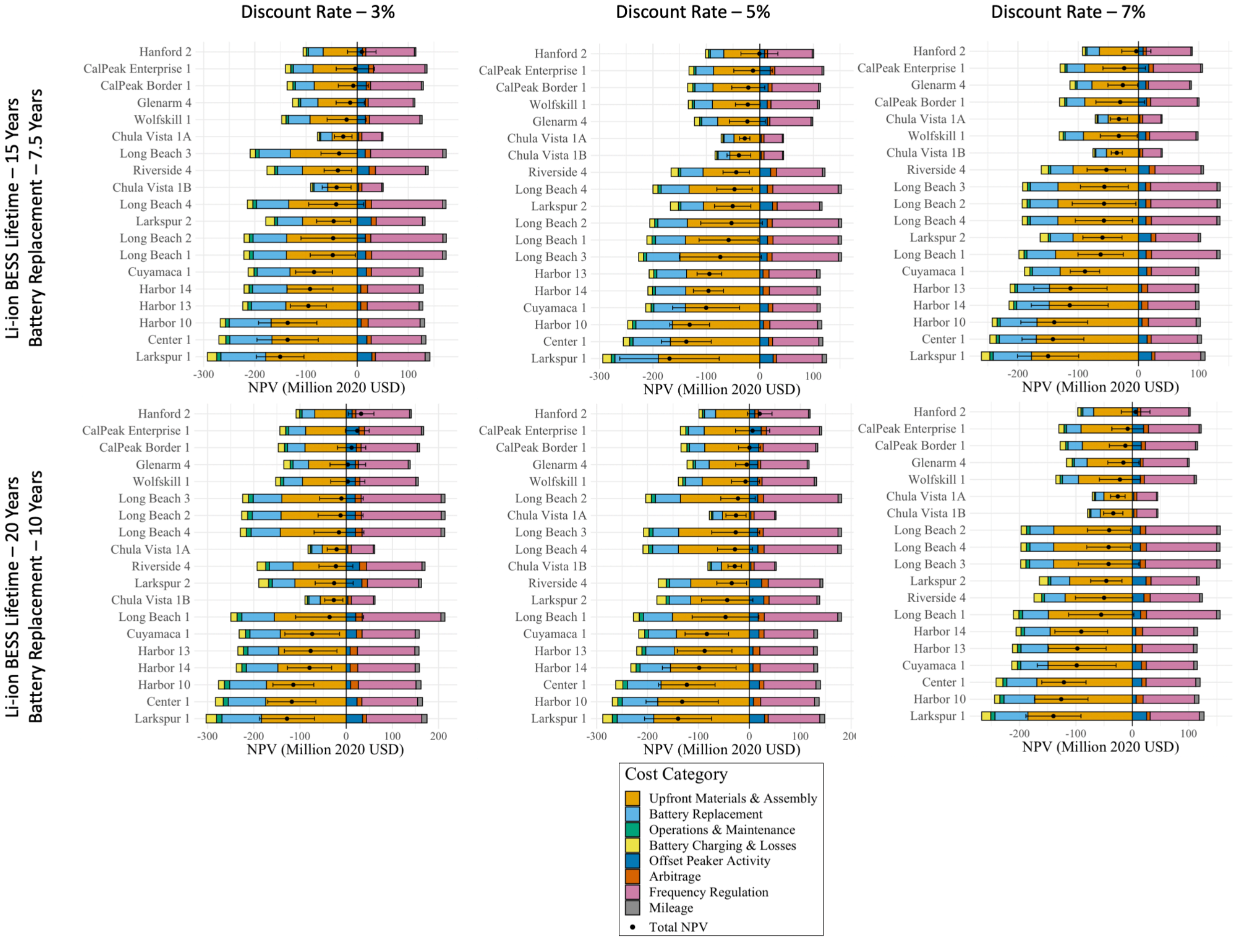

Figure S7: Net present values of BESS with NCA cathode chemistry and a social cost of carbon of \$51/tonne CO_{2eq}.

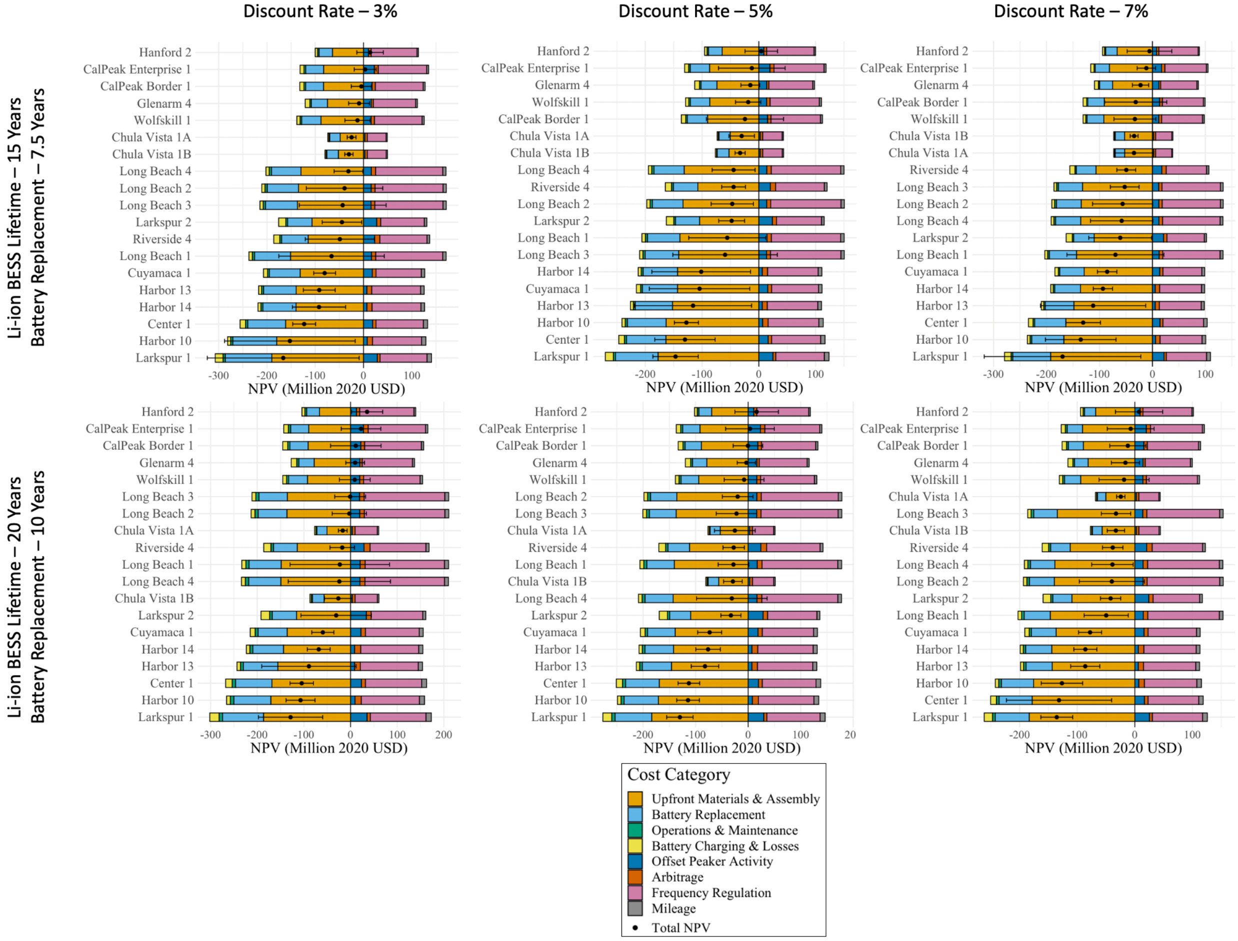

Figure S8: Net present values of BESS with NCA cathode chemistry and a social cost of carbon of $185/tonne CO_{2eq}.

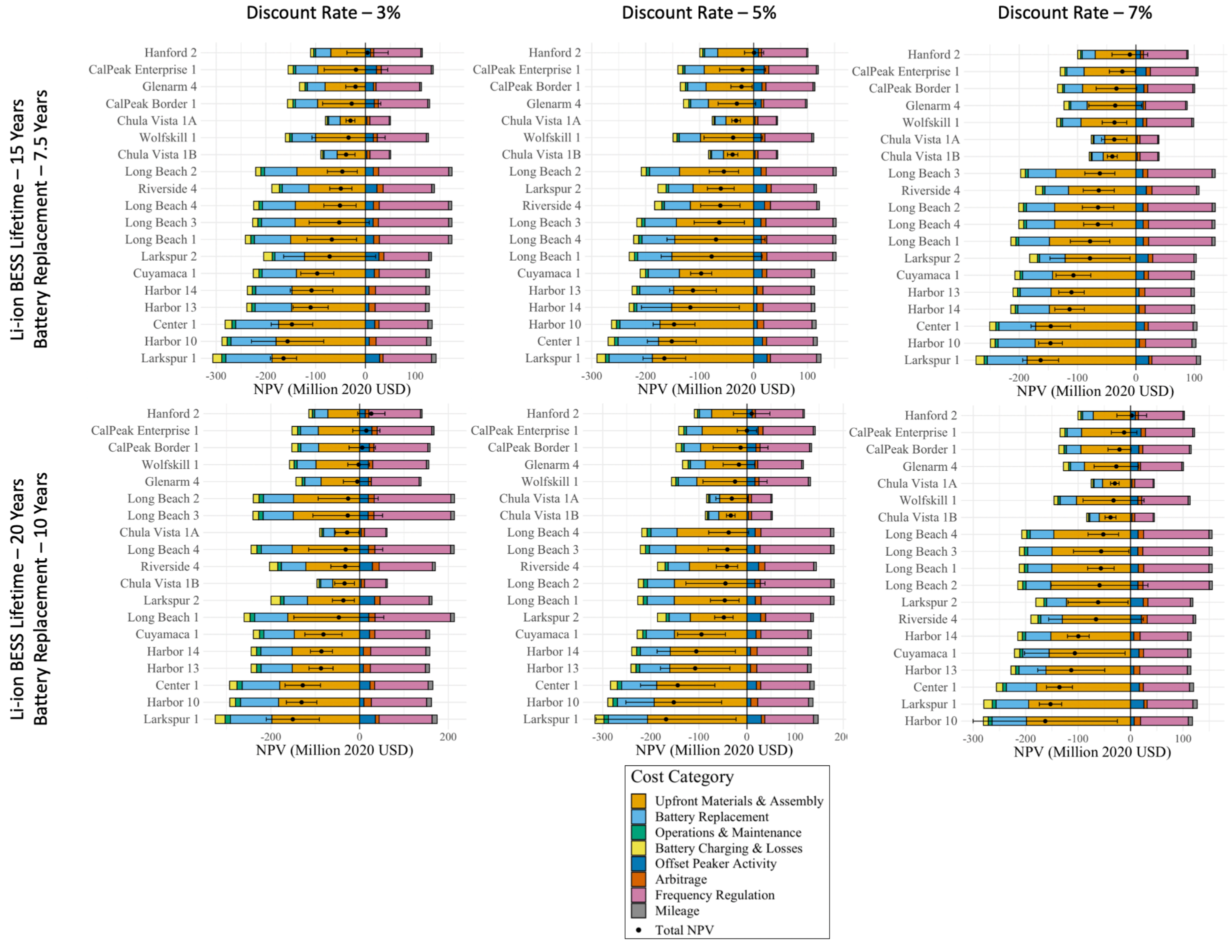

Figure S9: Net present values of BESS with NMC cathode chemistry and a social cost of carbon of \$51/tonne CO_{2eq}.

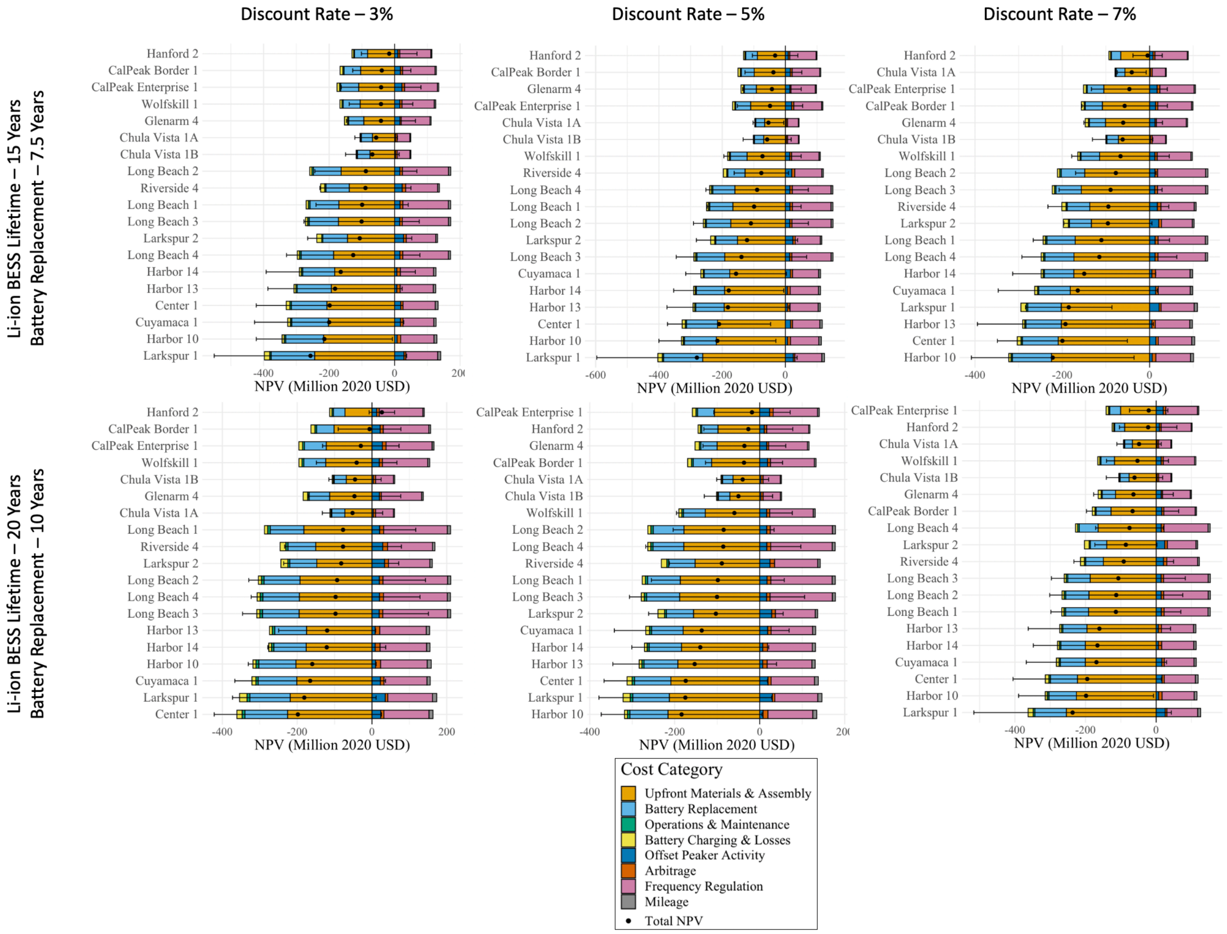

Figure S10: Net present values of BESS with NMC cathode chemistry and a social cost of carbon of \$185/tonne CO_{2eq}.

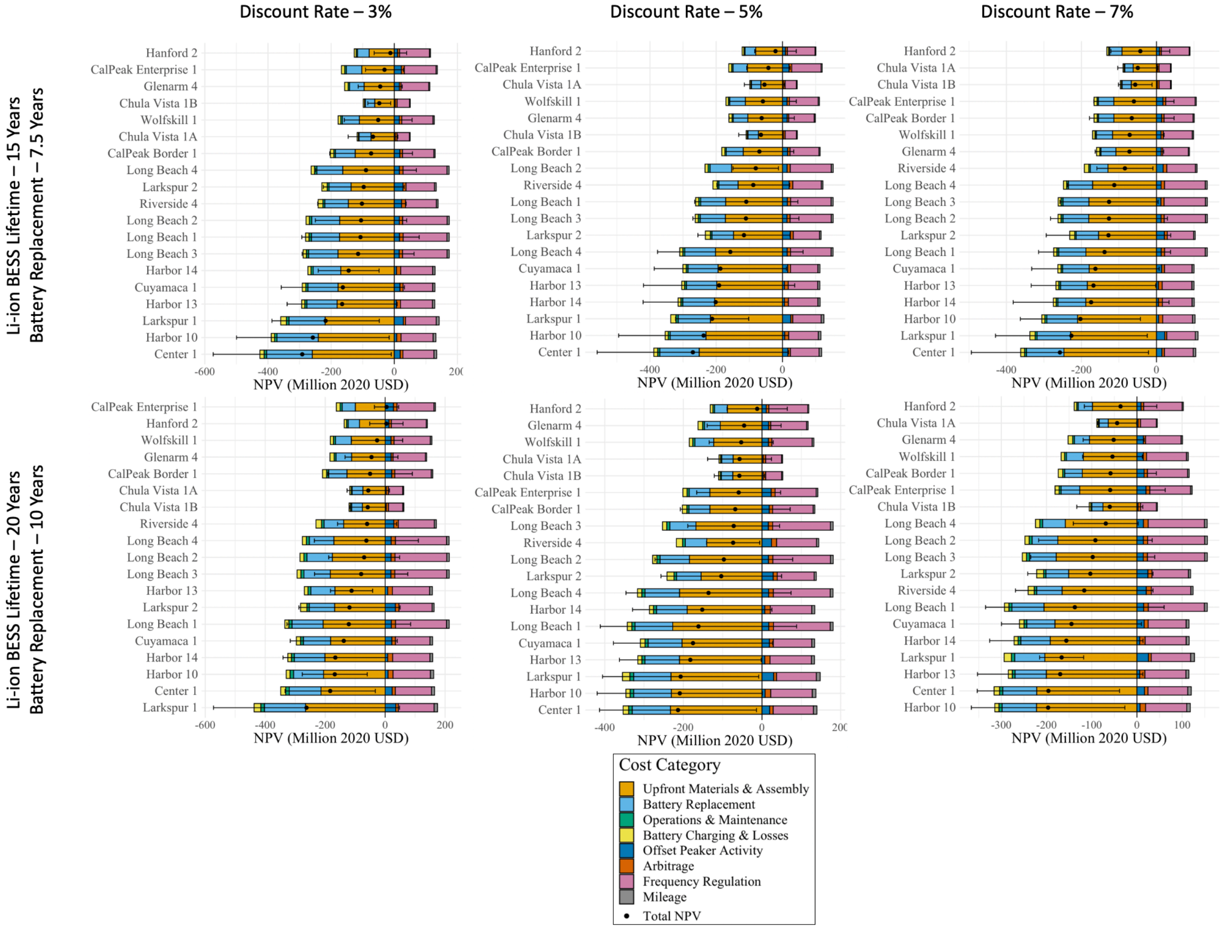

Figure S11a: Example LCA GWP of BESS replacing long beach generating station unit 1.

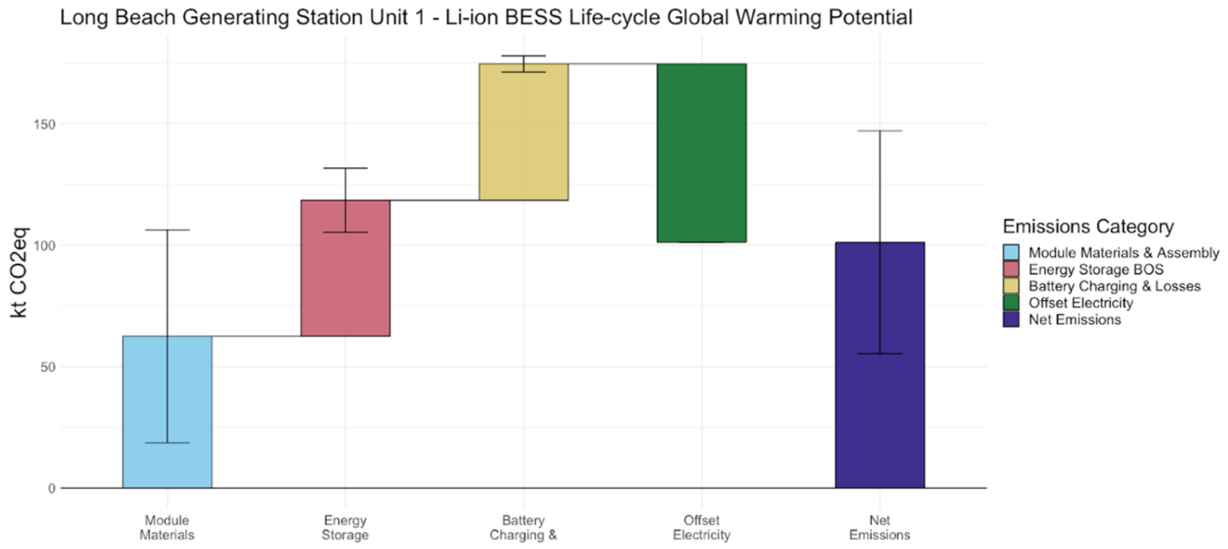

Figure S11b: Example LCA GWP breakdown of BESS replacing long beach generating station unit 1.

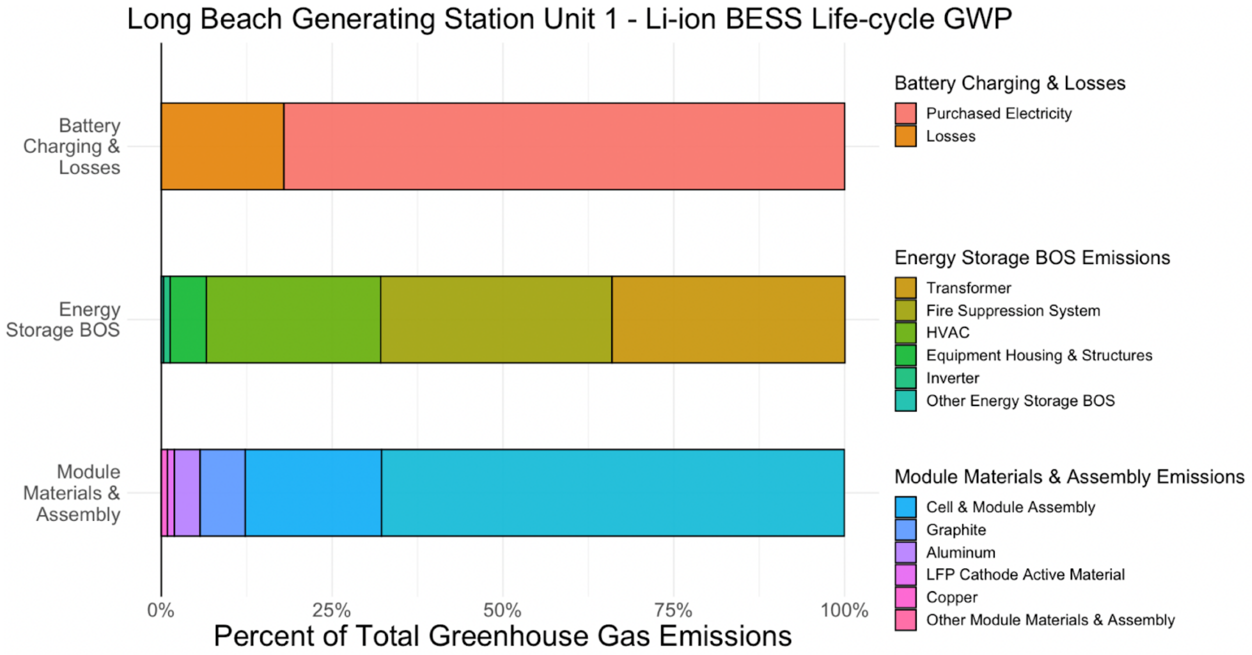

Exemplary graphical representation of the GWP of BESS replacing Long Beach Generating Station Unit 1. Emissions are categorized into "Battery Charging & Losses", "Energy Storage BOS Emissions", and "Module Materials & Assembly Emissions." A percent breakdown of the components contributing to each category are also presented.

Table S14: Distributions and assumptions for cell assembly and BOS of BESS.[18,45–47]

**Component/Parameter**	**Distribution/equation/description (prices and efficiencies)**
Battery Assembly Energy Demand (MJ/kWh) – Pilot Scale	Triangular distribution; mode = 1,000; min = 100; max = 10,000
Battery Assembly Energy Demand (MJ/kWh) – Nth Scale	Uniform distribution; min = 100; max = 1,000
Battery Assembly Energy Demand (MJ/kWh) - Unspecified	Triangular distribution; mode = 101; min = 100; max = 10,000
Battery Assembly Energy Supply – Max Thermal	Thermal energy percent of total energy = 80%<br>Electrical energy percent of total energy = 20%
Battery Assembly Energy Supply – Min Thermal	Thermal energy percent of total energy = 0%<br>Electrical energy percent of total energy = 100%
Battery Assembly Energy Supply – Unspecified Thermal	Thermal energy percent of total energy: uniform distribution; min = 0%, max = 80%<br>Electrical energy percent of total energy = 100% - thermal energy percent
Battery Assembly Thermal Source – Natural Gas	Thermal energy provided by natural gas = 100%
Battery Assembly Thermal Source – Coal	Thermal energy provided by coal = 100%
Battery Assembly Thermal Source – Unspecified	Thermal energy provided by natural gas: uniform distribution;<br>min = 0%, max = 100%<br>Thermal energy provided by coal = 100% - natural gas percent

For all runs presented in the main body of literature, the “unspecified” distributions for battery assembly energy demand, battery energy supply, and battery assembly thermal source are selected to include all possible scenarios for cell assembly.

Figure S12: Lifecycle greenhouse gas emissions of BESS with LFP cathode chemistry. Plants listed in order of highest to lowest expected NPV with a 3% discount rate and a social cost of carbon of \$51/tonne CO_{2eq}.

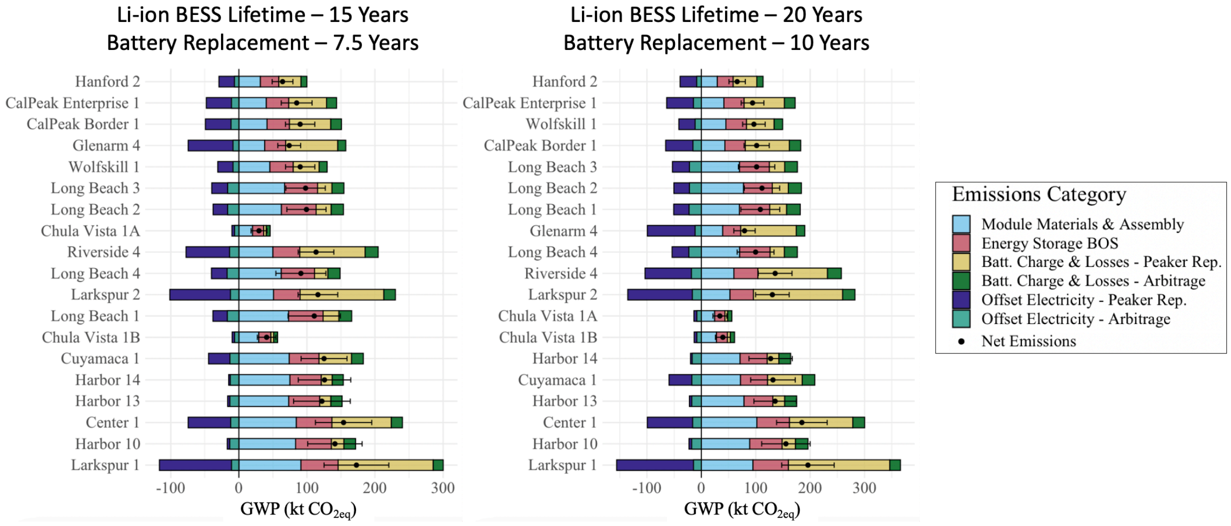

Figure S13: Lifecycle greenhouse gas emissions of BESS with NCA cathode chemistry. Plants listed in order of highest to lowest expected NPV with a 3% discount rate and a social cost of carbon of \$51/tonne CO_{2eq}.

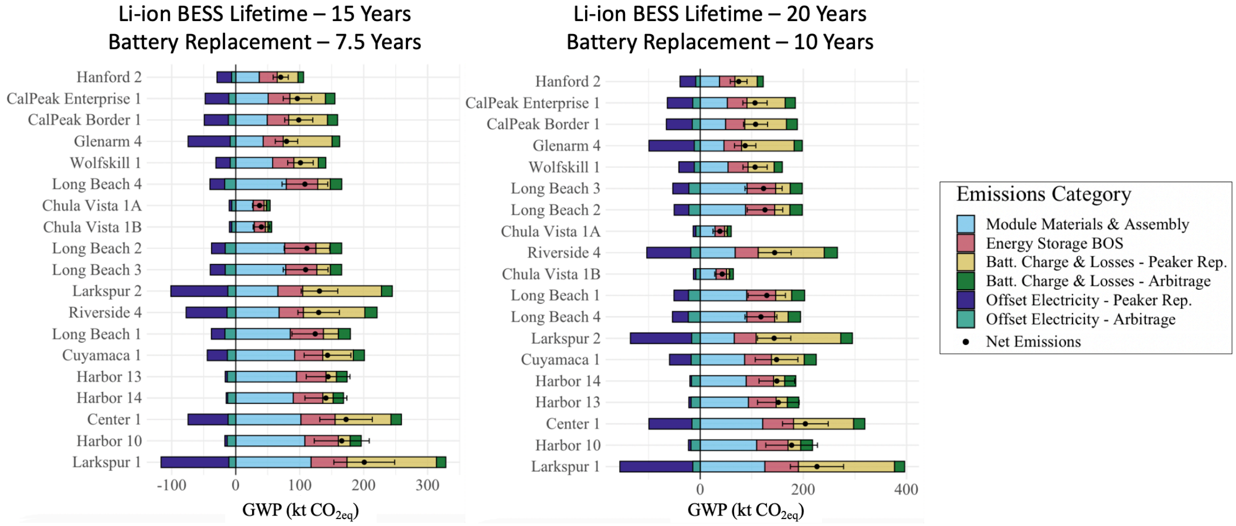

Figure S14: Lifecycle greenhouse gas emissions of BESS with NMC cathode chemistry. Plants listed in order of highest to lowest expected NPV with a 3% discount rate and a social cost of carbon of \$51/tonne CO_{2eq}.

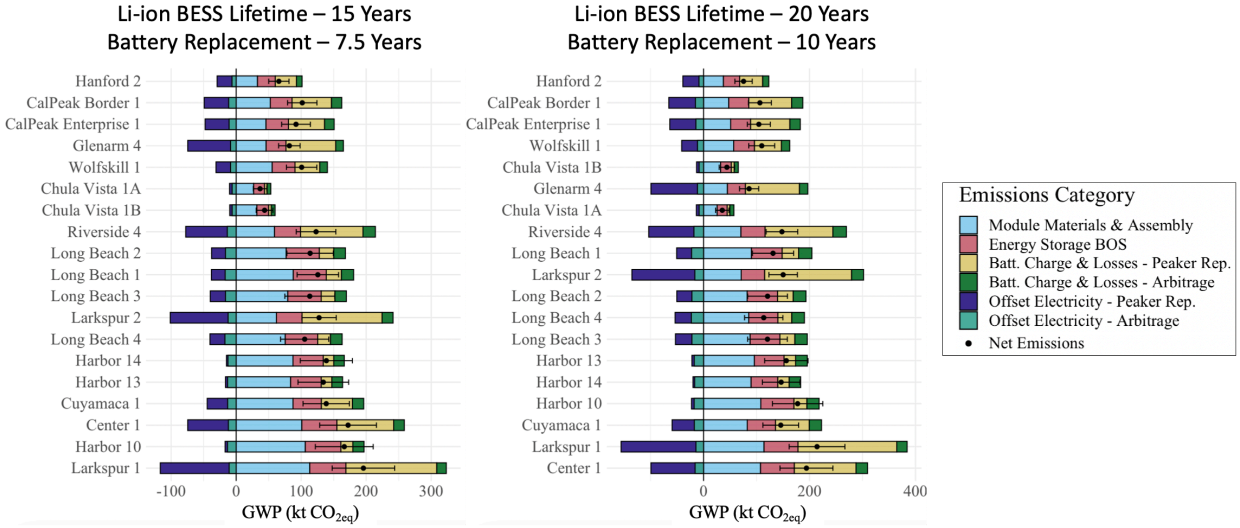

Table S15: Cost per cycle by Li-ion cathode chemistry derived from Monte Carlo Assessment.

Cathode Chemistry	Average Cost per Cycle [$/(MWh*Cycle)]	Standard Deviation
NMC	149.6	27.9
NCA	89.58	11.3
LFP	65.27	5.18

Cost per cycle is representative of the additional upfront cost associated with the increasing upfront battery capacity in order to compensate for the degradation that will occur from performing a cycle during the lifetime of a BESS. The impacts of non-cycle degradation are excluded from the cost per cycle. It is derived through a Monte Carlo simulation with 1000 runs that considers the degradation mechanisms and battery prices used throughout this study.

Table S16: Distributions and assumptions for BESS component LCAs.

**Component/Parameter**	**Distributions and characteristics**	**Source**
Li-ion Modules	<u>Material Breakdown:</u><br>Module material breakdown by chemistry taken from Porzio and Scown 2021 Table 3.	[18,48]
Inverters	<u>Mass Equation:</u><br>Mass [kg] = 1987.3 * Size [MW] ^ 0.7484<br><br><u>Material Breakdown by Mass:</u><br>Steel = 60.24%<br>Aluminum = 17.74%<br>Copper = 12.40%<br>Plastics = 9.62%<br><br><u>Energy Consumption:</u><br>Electricity [kWh] = 5.6856 * Size [MW] ^ 0.68<br>Refined Oil [MJ] = 0.1211 * Size [MW] ^ 0.68<br>Natural Gas [MJ] = 1.9131 * Size [MW] ^ 0.68<br>Heatwaste [MJ] = 4.9277 * Size [MW] ^ 0.68<br><br><u>Additional Characteristics:</u><br>Lifetime = 10 years	[49]

Transformers (Medium Voltage)	Mass Equation:<br>Mass [kg] = 2780.7 * Size [MW] ^ 0.8674<br><br>Material Breakdown by Mass:<br>Transformer oil = 24%<br>Steel = 56%<br>Copper = 12%<br>Pressboard = 3%<br>Paper = 1%<br>Other = 4%<br><br>Energy Consumption:<br>Electricity [kWh] = 1993.3 * Size [kW]<br>Natural Gas [kWh] = 3865.2 * Size [kW]<br><br>Additional Characteristics:<br>Lifetime = 40 years	[49,50]

Switchgears	<u>Mass:</u><br>Mass[kg] = 2400<br><br><u>Material Breakdown by Mass:</u><br>Aluminum = 15%<br>Steel = 31.67%<br>Stainless steel = 1.7917%<br>Copper = 1.0833%<br>SF_6 = 0.4167%<br>EPDM = 0.05%<br>Copper tungsten = 0.125%<br>PTFE = 0.0625%<br>Epoxy resin = 0.1667%<br>Molecular sieve = 0.070833%<br>Porcelain = 44.1667%<br>Other = 5.4%<br><br><u>Energy Consumption (Modeled as inverter):</u><br>Electricity [kWh] = 1993.3 * Size [kW]<br>Natural Gas [kWh] = 3865.2 * Size [kW]<br><br><u>Additional Characteristics:</u><br>SF_6 annual leak rate = 2%<br>Lifetime = 40 years	[49,51]
Conductors	<u>Mass Breakdown:</u><br>XLPE [kg/ft] = 0.1643 (XLPE modeled as HDPE)<br>Copper [kg/ft] = 0.887	[28]<br><br>Sampled from available products
Conduits	<u>Mass Breakdown:</u><br>Galvanized steel [kg/ft] = 2.1098	[28]<br><br>Sampled from available products

Battery Management System (BMS)	<u>Mass Breakdown:</u><br>Copper = 8.79%<br>Glass fiber = 9.99%<br>Epoxy resin = 3.03%<br>Steel = 43.0%<br>Aluminum = 4.53%<br>Nylon = 4.21%<br>HDPE = 4.57%<br>PC = 8.39%<br>Other = 13.5%<br><br><u>Energy Consumption:</u><br>Electricity [kWh] = 1.0275<br>Natural gas [MJ] = 0.03661	[52]
Fire Suppression	<u>Mass Breakdown (40 ft Container):</u><br>Galvanized steel = 635.81 [kg per container]<br>HFC227ea = 193.09 [kg per container]<br><br><u>Mass Breakdown (20 ft Container):</u><br>Galvanized steel = 408.74 [kg per container]<br>HFC227ea = 96.55 [kg per container]<br><br><u>Mass Breakdown (Cabinet):</u><br>Galvanized steel = 100 [kg per container]<br>HFC227ea = 25 [kg per container]<br><br><u>Additional Characteristics:</u><br>HFC227ea recovery leak rate: uniform distribution; min = 2.5%, max = 7.5%<br>HFC227ea lifetime = 10 years	[28,29,53–55]<br><br>Sampled from available products

HVAC	<u>Mass Breakdown (40 ft Container):</u><br>Steel = 156 [kg per container]<br>Galvanized steel = 70 [kg per container]<br>Aluminum = 34 [kg per container]<br>Copper = 34 [kg per container]<br>R410a: uniform distribution; min = 6; max = 9 [kg per container]<br><br><u>Mass Breakdown (20 ft Container):</u><br>Steel = 78 [kg per container]<br>Galvanized steel = 35 [kg per container]<br>Aluminum = 17 [kg per container]<br>Copper = 17 [kg per container]<br>R410a: uniform distribution; min = 3; max = 4.5 [kg per container]<br><br><u>Mass Breakdown (Cabinet):</u><br>(Assumed glycol cell cooling with negligible mass)<br><br><u>Additional Characteristics:</u><br>R410a annual leak rate: Triangular distribution; min = 2%; max = 20%, mode = 10%<br>R410a recovery leak rate: Triangular distribution; min = 15%; max = 100%; mode = 0.56%<br>HVAC lifetime = 15<br>R410a to R32 conversion = 1 to uniform distribution; min = 0.7; max = 0.8	[40–42,56]<br><br>See Table S17

Battery Container	Mass Breakdown (40 ft Container):<br>Steel = 3563 [kg per container]<br>Wood = 637.1 [kg per container]<br><br>Mass Breakdown (20 ft Container):<br>Steel = 1881 [kg per container]<br>Wood = 318.6 [kg per container]<br><br>Mass Breakdown (Cabinet):<br>Steel = 212.7 [kg per container]	Sampled from available products
Inverter Housing	Mass Breakdown (20 ft Container):<br>Steel = 1881 [kg per container]<br>Wood = 318.6 [kg per container]<br><br>Mass Breakdown (Cabinet):<br>Steel = 212.7 [kg per container]<br><br>Mass Breakdown (In Module):<br>No additional inverter housing	Sampled from available products

Foundation	Energy Consumption:<br>Electricity [MJ/cu-yard] = 0.01543<br>Residual Oil [MJ/cu-yard] = 0.0004071<br>Diesel [MJ/cu-yard] = 0.001750<br>Petcoke [MJ/cu-yard] = 0.01847<br>Natural Gas [MJ/cu-yard] = 0.01805<br>Coal [MJ/cu-yard] = 0.04213<br>Waste heat [MJ/cu-yard] = 0.004776<br>Tire [MJ/cu-yard] = 0.004776<br>Waste Oil [MJ/cu-yard] = 0.000742866<br>Renewables [MJ/cu-yard] = 0.0007428<br><br>Process Emissions:<br>CO2 [kg/cu-yard] = 0.07925<br>CH4 [kg/cu-yard] = $4.496 * 10^{-6}$<br>N2O [kg/cu-yard] = $4.62*10^{-7}$<br>VOC [kg/cu-yard] = $7.782*10^{-5}$<br>CO [kg/cu-yard] = $1.246*10^{-4}$<br>NOx [kg/cu-yard] = $1.415*10^{-4}$<br>PM10 [kg/cu-yard] = $7.408*10^{-5}$<br>PM25 [kg/cu-yard] = $2.566*10^{-5}$<br>SOx [kg/cu-yard] = $3.114*10^{-5}$	[48]
Battery Racks	Mass Breakdown (40 ft Container):<br>Steel: Uniform distribution; min = 1,200; max = 4,800 [kg per container]<br><br>Mass Breakdown (20 ft Container):<br>Steel: Uniform distribution; min = 600; max = 2,400 [kg per container]<br><br>Mass Breakdown (Cabinet):<br>Steel = 0 [kg per container]	Sampled from available products

Impacts from electricity consumption as associated with the US national average generation mix.

Table S17: HVAC Properties, Assumptions, and Calculations for BESS Cooling Load and Refrigerant Quantities.[40–42,57]

**Design Parameter/Assumptions**	**20 ft Container**	**40 ft Container**
Internal Thermal Load	9 kW<br>2.6 tons cooling<br>30,857 btu/hr	18 kW<br>5.1 tons cooling<br>61,714 btu/hr
Footprint	12 x 20 ft	12 x 40 ft
Height	10 ft	10 ft
U Value	0.1	0.1
Indoor Temp	70	70
Design Day Outdoor Temp	85	85
**Datacenter Modeling Approach**		
Oversizing factor	30%	30%
Environmental Thermal Load [btu/hr] (Roof & Wall Area * U Value * DT)	1320	2280
Total Load [btu/hr] (100% + Oversize Factor) * Internal Thermal Load + Environmental Thermal Load	41,194	82,509
Total Load [tons]	~3.33	~6.66
lbs of r410a per ton	Uniform distribution: min = 2 lbs per ton; max = 3 lbs per ton	Uniform distribution: min = 2 lbs; max = 3 lbs
Total r410a	Min = 6.66 lbs; max = 10 lbs	Min = 13.33 lbs; max = 20 lbs

References:

1. Bolun Xu, Dvorkin, Y., Kirschen, D. S., Silva-Monroy, C. A. & Watson, J.-P. A comparison of policies on the participation of storage in U.S. frequency regulation markets. in *2016 IEEE Power and Energy Society General Meeting (PESGM)* 1–5 (IEEE, 2016). doi:10.1109/PESGM.2016.7741531.

2. Fu, R., Remo, T. & Margolis, R. *2018 U.S. Utility-Scale Photovoltaics Plus-Energy Storage System Costs Benchmark*. (2018).

3. Ardani, K., O'Shaughnessy, E., Fu, R., McClurg, C., Huneycutt, J., Margolis, R. *Installed Cost Benchmarks and Deployment Barriers for Residential Solar Photovoltaics with Energy Storage: Q1 2016*. (2017).

4. Mongird, K., Viswanathan, V., Alam, J., Vartanian, C., Sprenkle, V., Baxter, R. *2020 Grid Energy Storage Technology Cost and Performance Assessment*. (2020).

5. Scown, C. D., Gokhale, A. A., Willems, P. A., Horvath, A. & McKone, T. E. Role of lignin in reducing life-cycle carbon emissions, water use, and cost for United States cellulosic biofuels. *Environ. Sci. Technol.* **48**, 8446–8455 (2014).

6. Yang, M., Baral, N. R., Anastasopoulou, A., Breunig, H. M. & Scown, C. D. Cost and life-cycle greenhouse gas implications of integrating biogas upgrading and carbon capture technologies in cellulosic biorefineries. *Environ. Sci. Technol.* **54**, 12810–12819 (2020).

7. Nordahl, S. L., Devkota, J. P., Amirebrahimi, J., Smith, S. J., Breunig, H. M., Preble, C. V., Satchwell, A. J., Jin, L., Brown, N. J, Kirchstetter T. W., Scown, C. D. Life-cycle greenhouse gas emissions and human health trade-offs of organic waste management strategies. *Environ. Sci. Technol.* **54**, 9200–9209 (2020).

8. Bird, R., Baum, Z. J., Yu, X. & Ma, J. The Regulatory Environment for Lithium-Ion Battery Recycling. *ACS Energy Lett.* **7**, 736–740 (2022).

9. Golmohammadzadeh, R., Faraji, F., Jong, B., Pozo-Gonzalo, C. & Banerjee, P. C. Current challenges and future opportunities toward recycling of spent lithium-ion batteries. *Renew. Sustain. Energy Rev* **159**, 112202 (2022).

10. Holland, S. P., Mansur, E. T., Muller, N. Z. & Yates, A. J. Are There Environmental Benefits from Driving Electric Vehicles? The Importance of Local Factors. *American Economic Review* **106**, 3700–3729 (2016).

11. Borenstein, S. & Bushnell, J. *Do two electricity pricing wrongs make a right? cost recovery, externalities, and efficiency*. (2018) doi:10.3386/w24756.

12. Graff Zivin, J. S., Kotchen, M. J. & Mansur, E. T. Spatial and temporal heterogeneity of marginal emissions: Implications for electric cars and other electricity-shifting policies. *J. Econ. Behav. Organ.* **107**, 248–268 (2014).

13. Davis, L. & Hausman, C. Market impacts of a nuclear power plant closure. *American Economic Journal: Applied Economics* **8**, 92–122 (2016).

14. Pacific Gas and Electric Company. The Next Giant Leap for Electric System Reliability: PG&E Proposes Nearly 1,600 MW of New Battery Energy Storage Capacity. *PG&E* https://www.pge.com/en_US/about-pge/media-newsroom/news-details.page?pageID=38883b6b-8597-4734-b85a-104a9f6e8af3&ts=1643133870903 (2022).

15. Wei, J. Interview on Utility-Scale Lithium-Ion Battery Energy Storage Cost Modeling. (2020).

16. Leggett, G. Interview on Lithium-Ion Utility-Scale Battery Energy Storage Cost Modeling. (2020).

17. Shriver, J. Interview on Lithium-Ion Utility-Scale Battery Energy Storage Cost Modeling. (2020).

18. Porzio, J. & Scown, C. D. Life-cycle assessment considerations for batteries and battery materials. *Adv. Energy Mater.* 2100771 (2021) doi:10.1002/aenm.202100771.

19. Bloch, C., Newcomb, J., Shiledar, S. & Tyson, M. *Breakthrough Batteries: Powering the Era of Clean Electrification*. (2020).

20. Mitchell, P. J. & Waters, J. E. *Energy Storage Roadmap Report*. (2017).

21. Ralon, P., Taylor, M., Ilas, A., Diaz-Bone, H. & Kairies, K.-P. *Electricity Storage and Renewables: Costs and Markets to 2030*. (2017).

22. Ecker, M., Nieto, N., Kabitz, S., Schmalstieg, J., Blanke, H., Warnecke, A., Sauer, D. U. Calendar and cycle life study of Li(NiMnCo)O2-based 18650 lithium-ion batteries. *J. Power Sources* **248**, 839–851 (2014).

23. Grolleau, S., Delaille, A., Gualous, H., Gyan, P., Revel, R., Bernard, J., Redondo-Iglesias, E., Peter, J. Calendar aging of commercial graphite/LiFePO4 cell – Predicting capacity fade under time dependent storage conditions. *J. Power Sources* **255**, 450–458 (2014).

24. Gantenbein, S., Schönleber, M., Weiss, M. & Ivers-Tiffée, E. Capacity Fade in Lithium-Ion Batteries and Cyclic Aging over Various State-of-Charge Ranges. *Sustainability* **11**, 6697 (2019).

25. Battaglia, V. Interview on Utility-Scale Lithium-Ion Battery Energy Storage Cost Modeling. (2020).

26. Srinivasan, V. Interview on Utility-Scale Lithium-Ion Battery Energy Storage Cost Modeling. (2020).

27. Fu, R., Feldman, D. & Margolis, R. *U.S. Solar Photovoltaic System Cost Benchmark: Q1 2018*. (2018).

28. RS Means 2018. *Building Construction Costs with RSMeans Data 2018*. vol. 76 (Gordian, 2018).

29. NFPA. NFPA 2001: Standard on Clean Agent Fire Extinguishing Systems. (2017).

30. NFPA. NFPA 855: Standard for the Installation of Stationary Energy Storage Systems. (2019).

31. Blodgett, D. & Hudson, R. *PV Inverter Useful Life Considerations*. (2019).

32. NFPA. NFPA 70: National Electric Code. (2019).

33. Bird, L., Flores, F., Volpi, C., Ardani, K., Manning, D., McAllister, R. *Review of Interconnection Practices and Costs in the Western States*. (2018).

34. Fritts, J. State and Local Sales Tax Rates, Midyear 2020. https://taxfoundation.org/state-and-local-sales-tax-rates-2020/ (2020).

35. Frith, J. *Lithium-Ion Batteries: State of the Industry 2021*. (2021).

36. Feldman, D., Ramasamy, V., Fu, R., Ramdas, A., Desai, J., Margolis, R. *U.S. solar photovoltaic system and energy storage cost benchmark: Q1 2020*. (2021) doi:10.2172/1764908.

37. Kang, J., Yan, F., Zhang, P. & Du, C. Comparison of comprehensive properties of Ni-MH (nickel-metal hydride) and Li-ion (lithium-ion) batteries in terms of energy efficiency. *Energy* **70**, 618–625 (2014).

38. Wood Mackenzie Power & Renewables. *The Global PV Inverter and PLPE Landscape, H1 2019*. (2019).

39. United States Code. *Subpart K - Distribution Transformers*. (2013).

40. US DOE. *Energy Conservation Program: Energy Conservation Standards for Single Package Vertical Air Conditioners and Single Package Vertical Heat Pumps*. (2015).

41. Gallagher, G., Despande, B., Gupta, P. & Huang, A. *California'sHigh Global Warming Potential Gases Emission Inventory - Emission Inventory Methodology and Technical Support Document*. (2016).

42. Karkour, S., Ihara, T., Kuwayama, T., Yamaguchi, K. & Itsubo, N. Life cycle assessment of residential air conditioners considering the benefits of their use: A case study in indonesia. *Energies* **14**, 447 (2021).

43. California ISO. California ISO Oasis. *Oasis Prod* http://oasis.caiso.com/mrioasis/logon.do.

44. California ISO. Today's Outlook. *California ISO* https://www.caiso.com/todaysoutlook/Pages/supply.html#section-batteries-trend.

45. Kurland, S. D. Energy use for GWh-scale lithium-ion battery production. *Environ. Res. Commun.* **2**, 012001 (2019).

46. Dai, Q., Kelly, J. C., Gaines, L. & Wang, M. Life Cycle Analysis of Lithium-Ion Batteries for Automotive Applications. *Batteries* **5**, 48 (2019).

47. Ellingsen, L. A.-W., Majeau-Bettez, G., Singh, B., Srivastava, A. K., Valoen, L. O., Stromman, A. H. Life Cycle Assessment of a Lithium-Ion Battery Vehicle Pack. *Journal of Industrial Ecology* **18**, 113–124 (2014).

48. Wang, M. *GREET2 Model*. (Argonne National Laboratory, 2020).

49. Rahman, M. M., Gemechu, E., Oni, A. O. & Kumar, A. The greenhouse gas emissions' footprint and net energy ratio of utility-scale electro-chemical energy storage systems. *Energy Conversion and Management* **244**, 114497 (2021).

50. Burger Mansilha, M., Brondani, M., Farret, F. A., Cantorski da Rosa, L. & Hoffmann, R. Life cycle assessment of electrical distribution transformers: comparative study between aluminum and copper coils. *Environ. Eng. Sci.* **36**, 114–135 (2019).

51. Bessede, J.-L. & Krondorfer, W. *Impact of High-voltage SF6 Circuit Breakers on Global Warming - Relative Contribution of SF6 Losses*. https://www.epa.gov/sites/default/files/2016-02/documents/conf00_krondorfer.pdf (2017).

52. Farzad, T. *Life Cycle Analysis for a DC- microgrid energy system in Fjärås*. (2019).

53. Kopylov, S. N., Kopylov, P. S., Eltyshev, I. P., Kopylov, N. P. & Begishev, I. R. Highly Effective Fire Extinguishing Mixtures of Iodinated and Fluorinated Hydrocarbons as a Way to Reduce Greenhouse Gas Emissions into the Atmosphere. *IOP Conf. Ser.: Earth Environ. Sci.* **666**, 022011 (2021).

54. Senecal, J. & Prescott, R. *FM-200 Suppression Systems: A Conservative Discharge Test Method and In-Room Pressure Variance Upon Discharge*. (1995).

55. BNP Media. Fire Protection Design with FM 200. *Plumbing and Mechanical Engineer* (2000).

56. ACR News. R32 shows positive benefits in R410A drop-in test. *ACR News* https://www.acr-news.com/r32-shows-positive-benefits-in-r410A-drop-in-test (2013).

57. Cubero, E. Communications with Edward Cubero. (2021).
